# High-quality chromosome-scale assembly of the walnut (*Juglans regia* L.) reference genome

**DOI:** 10.1093/gigascience/giaa050

**Published:** 2020-05-20

**Authors:** Annarita Marrano, Monica Britton, Paulo A Zaini, Aleksey V Zimin, Rachael E Workman, Daniela Puiu, Luca Bianco, Erica Adele Di Pierro, Brian J Allen, Sandeep Chakraborty, Michela Troggio, Charles A Leslie, Winston Timp, Abhaya Dandekar, Steven L Salzberg, David B Neale

**Affiliations:** 1 Department of Plant Sciences, University of California, Davis, One Shields Avenue, Davis, CA 95616, USA; 2 Bioinformatics Core Facility, Genome Center, University of California, One Shields Avenue, Davis, CA 95616, USA; 3 Department of Biomedical Engineering, Johns Hopkins University, 720 Rutland Avenue, Baltimore, MD 21205, USA; 4 Center for Computational Biology, Whiting School of Engineering, Johns Hopkins University, 3100 Wyman Park Dr., Baltimore, MD 21211, USA; 5 Research and Innovation Center, Fondazione Edmund Mach, Via E. Mach, 1 38010 S. Michele all'Adige (TN) 38010, Italy; 6 Departments of Computer Science and Biostatistics, Johns Hopkins University, 3400 North Charles Street Baltimore, MD 21218, USA

**Keywords:** Nanopore, Hi-C, Iso-Seq, gene prediction, genetic diversity, proteome, allergens

## Abstract

**Background:**

The release of the first reference genome of walnut (*Juglans regia* L.) enabled many achievements in the characterization of walnut genetic and functional variation. However, it is highly fragmented, preventing the integration of genetic, transcriptomic, and proteomic information to fully elucidate walnut biological processes.

**Findings:**

Here, we report the new chromosome-scale assembly of the walnut reference genome (Chandler v2.0) obtained by combining Oxford Nanopore long-read sequencing with chromosome conformation capture (Hi-C) technology. Relative to the previous reference genome, the new assembly features an 84.4-fold increase in N50 size, with the 16 chromosomal pseudomolecules assembled and representing 95% of its total length. Using full-length transcripts from single-molecule real-time sequencing, we predicted 37,554 gene models, with a mean gene length higher than the previous gene annotations. Most of the new protein-coding genes (90%) present both start and stop codons, which represents a significant improvement compared with Chandler v1.0 (only 48%). We then tested the potential impact of the new chromosome-level genome on different areas of walnut research. By studying the proteome changes occurring during male flower development, we observed that the virtual proteome obtained from Chandler v2.0 presents fewer artifacts than the previous reference genome, enabling the identification of a new potential pollen allergen in walnut. Also, the new chromosome-scale genome facilitates in-depth studies of intraspecies genetic diversity by revealing previously undetected autozygous regions in Chandler, likely resulting from inbreeding, and 195 genomic regions highly differentiated between Western and Eastern walnut cultivars.

**Conclusion:**

Overall, Chandler v2.0 will serve as a valuable resource to better understand and explore walnut biology.

## Introduction

Persian walnut (*Juglans regia* L.) is among the top 3 most-consumed nuts in the world, and over the past 10 years, its global production increased by 37% [1]. Its richness in alpha-linolenic acid (ALA), proteins, minerals, and vitamins, along with documented benefits for human health, explains this increased interest in walnut consumption [2]. As suggested by its generic name *Juglans* from the Latin appellation “*Jovis glans*,” which loosely means “nut of gods,” the culinary and medical value of Persian walnut was already widely prized by ancient civilizations [3].

The origin and evolution of the Persian walnut are the results of a complex interplay between hybridization, human migration, and biogeographical forces [4]. A recent phylogenomic analysis revealed that Persian walnut (and its landrace *Juglans sigillata*) arose from an ancient hybridization that occurred between American black walnuts and Asian butternuts after a climate-driven range expansion in Eurasia during the Pliocene [5]. Evidence suggests that the mountains of Central Asia were the cradle of domestication of Persian walnut [6], whence it spread to the rest of Asia, the Balkans, Europe, and, finally, the Americas.

Today, walnut is cultivated worldwide in an area of 1,587,566 ha, mostly in China and the USA [7]. Considerable phenotypic and genetic variability can be observed in this wide distribution area, especially in the Eastern countries, where walnuts can still be found in wild fruit forests. Many studies on genetic diversity in walnut have outlined a genetic differentiation between Eastern and Western genotypes [8, 9]. Moreover, walnuts from Eastern Europe, Central Asia, and China exhibit higher genetic diversity and a higher number of rare alleles than the genotypes from Western countries [10].

The release of the first reference genome, Chandler v1.0 [11], enabled the study of walnut genetics at a genome-wide scale. For the first time, it was possible to explore the gene space of Persian walnut with the prediction of 32,498 gene models, providing the basis to untangle complex phenotypic pathways, such as those responsible for the synthesis of phenolic compounds. The availability of a reference genome marked the beginning of a genomics phase in Persian walnut, allowing whole-genome resequencing [5, 12], the development of high-density genotyping tools [9, 13], and the genetic dissection of important agronomical traits in walnut [14–17]. However, the Chandler v1.0 assembly is highly fragmented, compromising the accuracy of gene prediction and the fulfillment of advanced genomics studies necessary to resolve many, still-unanswered questions in walnut research.

The recent introduction of long-read sequencing technologies and long-range scaffolding methods has enabled chromosome-scale assembly for multiple plant species, including highly heterozygous crops such as almond (*Prunus dulcis* [18]) and kiwifruit (*Actinidia eriantha* [19]). The availability of genomes with fully assembled chromosomes provides foundations for understanding plant domestication and evolution [18, 20, 21] and the mechanisms governing important traits (e.g., flower color and scent [22]), as well as the impact of epigenetic modifications on phenotypic variability [23]. Recently, Zhu et al. [24] assembled the parental genomes of a hybrid *Juglans microcarpa* × *J. regia* (cv. Serr) at the chromosome scale using long-read Pacific Biosciences (PacBio) sequencing and optical mapping. They relied on the haplotype divergence between the 2 *Juglans* species and demonstrated an ongoing asymmetric fractionation of the 2 subgenomes present in *Juglans* genomes.

Here we report a new chromosome-level assembly of the walnut reference genome, Chandler v2.0, which we obtained by combining Oxford Nanopore long-read sequencing [25] with chromosome conformation capture (Hi-C) technology [26]. Thanks to the increased contiguity of Chandler v2.0, we were able to substantially improve gene prediction accuracy, with new, longer gene models identified and many fewer artifacts compared to Chandler v1.0. Also, the availability of full, chromosomal sequences reveals new genetic diversity of Chandler, previously inaccessible through standard genotyping tools, and significant genetic differentiation between Western and Eastern walnuts at 195 genomic regions, including also loci involved in nut shape and harvest date. In the present research, we demonstrate the fundamental role of a chromosome-scale reference genome to integrate transcriptomics, population genetics, and proteomics, which in turn enable a better understanding of walnut biology.

## Results

### Genome long-read sequencing and assembly

To increase the contiguity of the Chandler genome, we first generated deep sequence coverage using Oxford Nanopore Technology (ONT), a cost-effective long-read sequencing approach that determines DNA bases by measuring the changes in electrical conductivity generated while DNA fragments pass a tiny biological pore [27]. Since the release of the first plant genome assembly generated using ONT sequencing [28], this technology has been applied to sequence and obtain chromosome-scale genomes of many other plant species [29–31]. In Persian walnut, ONT sequencing yielded 7,096,311 reads that provided 21.9 Gb of sequence, or ∼35× genome coverage (assuming a genome size of 620 Mb). Read lengths averaged 3.1 kb, and the N50 read length was 6.7 kb, with the longest read being 992.2 kb (Supplementary Table S1).

One of the major limitations of long-read sequencing technologies is their high error rate, which can range between 5% and 15% for Nanopore sequencing [32]. To overcome this limitation, we adopted the hybrid assembly technique incorporated into the MaSuRCA assembler, which combines long, high-error reads with shorter but much more accurate Illumina sequencing reads to generate a robust, highly contiguous genome assembly [33]. First, using the Illumina reads, we created 3.7 million “super-reads” with a total length of 2.9 Gb. We then combined the super-reads with the ONT reads to generate 3.2 million mega-reads with a mean length of 4.7 kb, representing 24× genome coverage (Supplementary Table S2). Finally, we assembled the mega-reads to obtain the “hybrid” Illumina-ONT assembly, which comprised 1,498 scaffolds, 258 contigs, and 25,007 old scaffolds from Chandler v1.0 (Supplementary Table S3).

Even though the total number of scaffolds (>1 kb) was reduced by 80% compared to Chandler v1.0 (Table 1), the new hybrid assembly was still fragmented. To improve the assembly further and build chromosome-scale scaffolds, we applied Hi-C sequencing, which is based on proximity ligation of DNA fragments in their natural conformation within the nucleus [26]. The HiRise scaffolding pipeline processed 356 million paired-end 100-bp Illumina reads to generate the HiRise assembly (Table 1). The top 17 scaffolds from this assembly spanned >90% of the total assembly length, with a scaffold length ranging from 19.6 to 45.2 Mb (Supplementary Figs S1 and S2). As reported in Table 1, the Chandler genome contiguity increased dramatically compared with the previous assemblies. Compared with the recently published genome assembly of the walnut cultivar Serr [24], Chandler v2.0 was less contiguous at the contig level, with an N50 size of 1.1 Mb against the 15.1 Mb of JrSerr_v1.0. The higher-coverage PacBio sequencing data (57.2 Gb) used to assemble JrSerr_v1.0 may explain this discrepancy in contiguity between the 2 assemblies. Besides, our assembly presented a value of contiguity similar to that of the recently published genomes of pecan (*Carya illinoinensis*; 1.1 Mb [34]), Chinese chestnut (*Castanea mollissima*; 944.4 kb [35]), and pedunculate oak (*Quercus robur*; 1.35 Mb [36]).

**Table 1:** Comparison among the 4 assemblies of Chandler

Statistic	Chandler v1.0	Chandler v1.5	Chandler hybrid	Chandler HiRise	Chandler v2.0	JrSerr_v1.0
No. of scaffolds	27,032	4,401	3,497	2,656	2,643	73
N50 length (scaffolds) (bp)	304,423	637,984	1,640,935	32,655,472	37,114,715	35,197,335
L50 (scaffolds)	344	272	89	8	7	7
Total length of assembled scaffolds (bp)	667,299,356	650,478,320	567,378,842	567,480,142	567,796,851	534,671,929
No. of contigs	53,156	7,411	3,592	3,700	3,684	127
N50 length (contigs) (bp)	42,417	317,751	1,512,354	1,083,883	1,083,883	15,066,219
L50 (contigs)	3,630	482	97	144	144	13
Total size of assembled contigs (bp)	641,521,787	617,088,256	567,276,004	567,276,244	567,192,099	530,618,363

Scaffolds shorter than 1,000 bp are not included in these totals.

### Validation of the HiRise assembly

**Figure 1:**
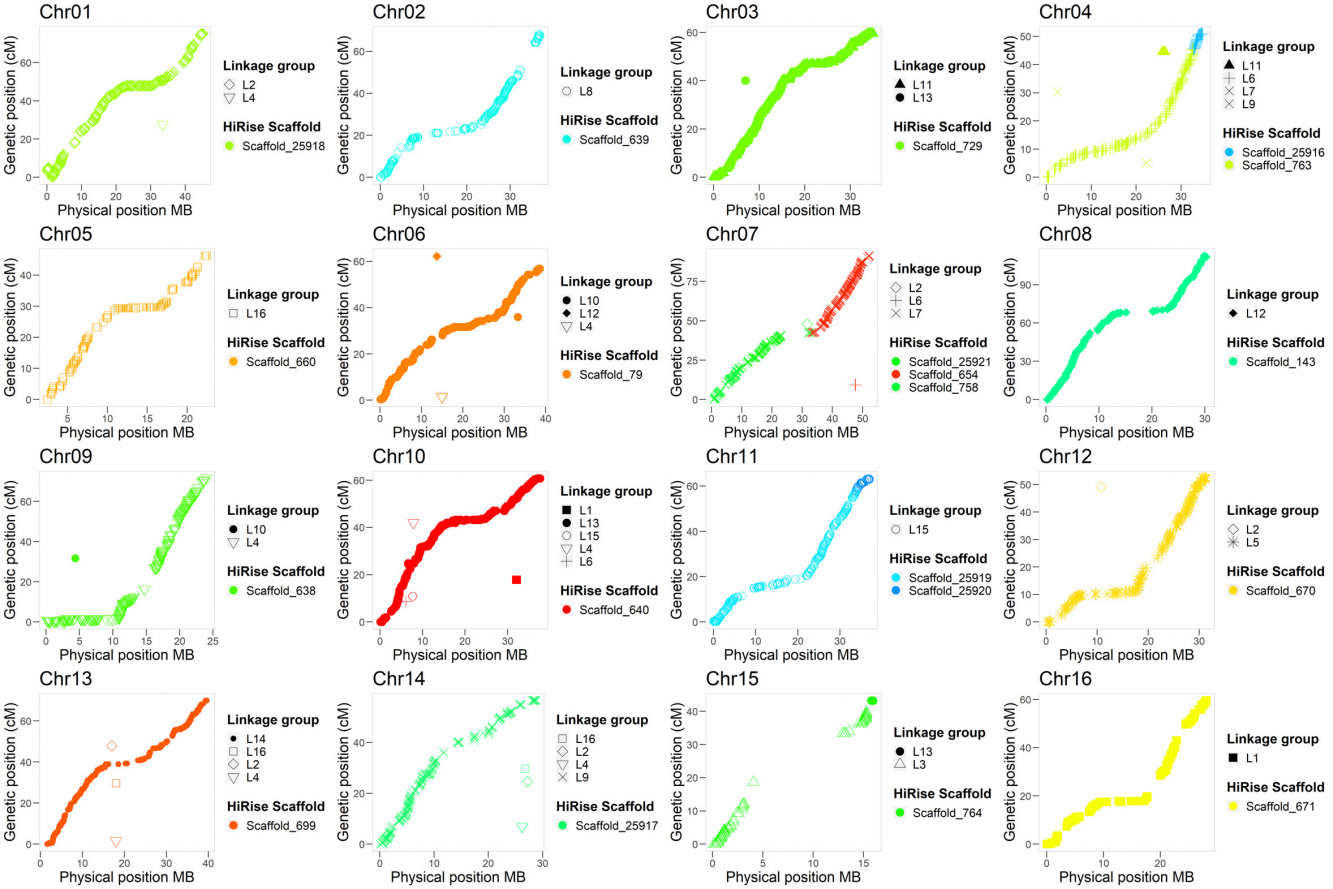
Collinearity between the high-density “Chandler” genetic map of Marrano et al. [16] and the 16 chromosomal pseudomolecules of Chandler v2.0.

To assess the quality of the HiRise assembly, we used 2 independent sources of data. First, we used the single-nucleotide polymorphism (SNP) markers mapped on the high-density genetic map of Chandler recently described by Marrano et al. [16]. Out of the 8,080 SNPs mapped into 16 linkage groups (LGs), 6,894 had probes aligning uniquely on the HiRise assembly, with 98% of identity for >95% of their length. A total of 35 scaffolds of the HiRise assembly could be anchored to a chromosomal LG by ≥1 SNP (Fig. 1). In particular, 13 LGs were spanned by a single HiRise scaffold, while 2–3 scaffolds each aligned the remaining 3 LGs.

Second, we anchored the HiRise assembly to the Chandler genetic map used by Luo et al. [37] to construct a walnut physical map. In total, 972 of the mapped markers (1,525 SNPs) aligned uniquely on the same 35 HiRise scaffolds anchored to the aforementioned linkage map. Overall, we observed almost perfect collinearity between the HiRise assembly and both Chandler genetic maps (Fig. 1, Supplementary Fig. S3). Therefore, we oriented, ordered, and named the HiRise scaffolds consistent with the linkage map of Luo et al. [37], generating the final 16 chromosomal pseudomolecules of *J. regia* Chandler.

**Figure 2:**
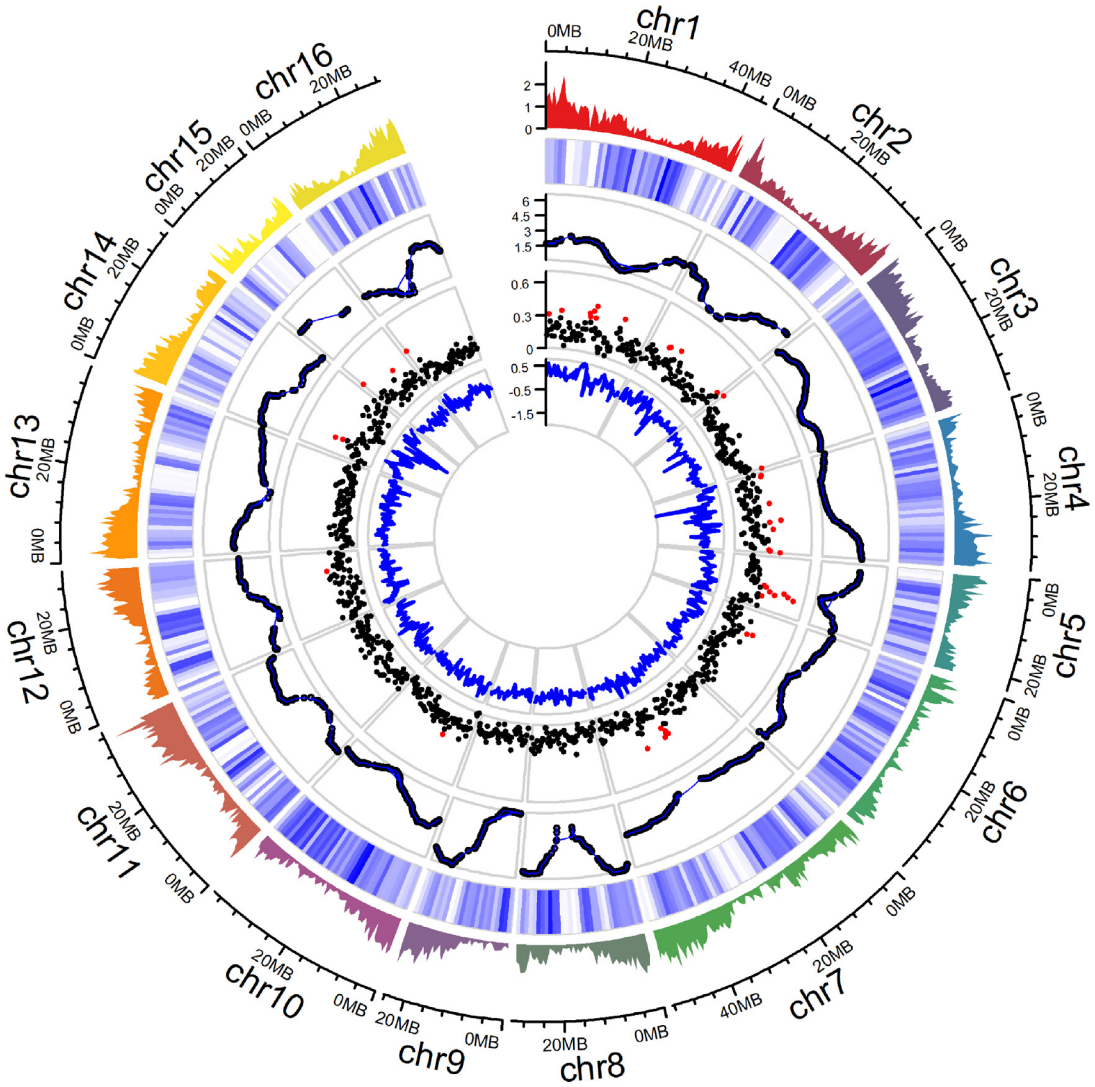
Summary of gene distribution and genetic diversity across the 16 chromosomes of Chandler v2.0. Tracks from outside to inside: (i) gene density of Chandler v2.0 in 1-Mb windows; (ii) Chandler heterozygosity in 1-Mb windows (white = low heterozygosity; blue = high heterozygosity); (iii) Recombination rate for sliding windows of 10 Mb (average = 2.63 cM/Mb); (iv) *F*_ST_ in 500-kb windows. Windows in the 95th percentiles of the *F*_ST_ distribution are highlighted in red; (v) ROD values for 500-kb windows.

These 16 contiguous chromosomal scaffolds account for 95% of the final walnut reference genome v2.0, with an N50 scaffold size of 37 Mb. We identified telomere sequences at both ends for 9 of the chromosome scaffolds, on 1 end of the other 7 chromosomes, and 1 end of 7 unanchored scaffolds. Also, all 16 chromosomes had centromeric repeats in the middle, alongside regions with low recombination rates (Fig. 2).

As compared to the previous Chandler genome assemblies (Table 1), Chandler v2.0 had a smaller genome size (573.9 Mb), much closer to the Genomescope estimate of 488.2 Mb. This reduction in genome size represents a great improvement of Chandler v2.0 and can be related to the removal of haplotype variants, likely interpreted and annotated as different scaffolds in the previous genome versions. Compared with the Serr walnut genome (JrSerr_v1.0; 534.7 Mb) [24], Chandler v2.0 had a larger genome size, likely due to structural variation (e.g., copy number and presence/absence variants), whose central role in explaining intraspecific genomic and phenotypic diversity has been reported in different plant species [38, 39]. In addition, the higher number of unanchored scaffolds (2,631; 20.9 Mb) in Chandler v2.0 compared with JrSerr_v1.0 can represent autozygous genomic regions of Chandler, devoid of segregating markers and, therefore, difficult to anchor to linkage genetic maps [37], as also suggested by the higher fixation index (*F*) observed in Chandler (0.03) than Serr (−0.29) in previous genetic surveys [9]. The 2 walnut assemblies, however, aligned with high sequence identity (>98% for >95% of their total length) and showed high collinearity (Supplementary Fig. S4). Future comparative genomics studies will provide further insights on the functional and structural differences between the 2 genome assemblies, and their explanatory involvement in the morphological and physiological variation of these 2 walnut cultivars.

To assess the sequence accuracy of Chandler v2.0, we first compared the scaffold sequences of Chandler v2.0 with the previous version of the walnut reference genome. Approximately 578 Mb of sequence were mutual best alignments, namely, best hits of each location between Chandler v2.0 and v1.0 and vice versa, with a sequence identity of 99.6%. We also observed that 135 Mb of Chandler v1.0 (18.9%) aligned to the same locations in Chandler v2.0, suggesting the presence of redundant haplotypes in the previous version of the walnut reference genome that have been removed in our assembly. We then mapped the Illumina whole-genome shotgun data [11] against the new chromosome-scale genome. The alignment resulted in 64,950,691,681 bp mapped, of which 407,450,406 were single-base mismatches, consistent with an Illumina sequence accuracy rate of 99.5%.

### Repeat annotation

More than half (58.4%) of the new Chandler v2.0 is repetitive. This estimate is higher than the previous version of the walnut reference genome (51.19%) and comparable to other *Fagales* genomes [36, 40]. As in most plant genomes, interspersed repeats were the most abundant type of repeats, with retrotransposons at 36.45% and DNA transposons at 15.86%. Gypsies (10.5%) and Copias (7.69%) were the most represented classes of long-terminal retrotransposons (LTR), and, though widely dispersed throughout the genome, they were distributed differently along the 16 chromosomes (Supplementary Fig. S5): the Gypsy LTRs were more abundant alongside the centromeres, where, instead, the density of the Copia LTRs decreased, as previously observed in walnut [24]. The long-interspersed nuclear elements (L1/LINE), which possess a poly(A) tail and 2 open reading frames for autonomous retrotransposition, were the largest class of non-LTRs, at 7.14% of the genome. Simple repeats (1.91%) were also found.

### PacBio Iso-Seq sequencing and gene annotation

A fragmented reference genome can severely hamper the accuracy of gene prediction because many genes will be broken across multiple small contigs (false-negative results) and because heterozygous gene variants may be annotated separately (false-positive results).

To improve the gene prediction accuracy of Chandler v2.0, we used the “Isoform Sequencing” (Iso-Seq) method, developed by PacBio, which can generate full-length transcripts up to 10 kb, allowing for accurate determination of exon-intron structure by the alignment of the transcripts to the assembly [41]. The high error rate of PacBio sequencing can be greatly reduced using circular consensus sequence (CCS), in which a transcript is circularized and then sequenced repeatedly to self-correct the errors. We applied PacBio Iso-Seq to sequence full-length transcripts from 9 tissues, chosen to cover most of the transcript diversity in walnut (Supplementary Table S4). Across the 4 SMRT cells, we obtained 26,328,087 subreads with a mean length of 1,188 bp (Supplementary Table S5) and CCSs ranging from 13,000 to 142,000 per library (Supplementary Table S6). Of the 745,730 full-length non-chimeric (FLnc) transcripts, 68,225 were classified as high quality, FL (HQ FL) consensus transcript sequences, with an average length of 1,357 bp (Supplementary Table S6). Catkin 1-inch elongated (CAT1), shoot, and root yielded the lowest number of HQ FL transcripts, while pollen and leaf had the lowest number of HQ consensus clusters obtained per CCS after polishing (Supplementary Table S6). These results can be explained by lower complementary DNA (cDNA) quality or fewer inserts of full-length transcripts from these tissues during the cDNA pooling and library preparation. Nevertheless, >99% of the HQ FL transcripts aligned onto the new chromosomal-level walnut reference genome (Supplementary Table S7).

By combining the HQ FL transcripts with available *Juglans* transcriptome sequences, we identified 37,554 gene models, which are more than those annotated in Chandler v1.0 but fewer than the predicted genes in the NCBI RefSeq *J. regia* annotation generated with the first version of the reference genome (Table 2). Thus, the new chromosome-scale genome, along with the availability of full-length transcripts, allowed us to identify genes missed in Chandler v1.0 due to genome fragmentation, as well as to remove false-positive predictions likely caused by heterologous variants of the same locus mistakenly interpreted and annotated as independent scaffolds in Chandler v1.0. Also, the mean gene length in Chandler v2.0 was higher than the previous gene annotations (Table 2), a consequence of the increased contiguity of the new chromosome-scale reference genome. The average gene density of Chandler v2.0 was 19.75 genes per 100 kb, with higher gene content in the proximity of telomeric regions (Fig. 2), consistent with other plant genomes [21, 42]. The majority of the predicted gene models of Chandler v2.0 were supported by expression data and showed high similarity with a protein-coding transcript of other plant species (Supplementary Table S8). Also, 30,318 models were annotated with 8,243 different Gene Ontology (GO) terms (Supplementary Figs S6–S8).

**Table 2:** Statistics on the gene annotation of Chandler v2.0 compared with the previous gene annotations of the Chandler genome

Statistics	Chandler v2.0	Chandler v1.0	Chandler RefSeq v1.0
No. of genes	37,554	32,496	41,188
Mean gene length (bp)	5,319	4,358	4,641
Single-exon transcripts	6,613	6,247	6,749
Mean CDS length (bp)	1,335	1,222	1,336
No. of exons	242,208	172,273	230,261
Mean exon length (bp)	257.8	229.5	314
No. of introns	201,290	139,775	181,419
Mean intron length	853.9	730	835
Mean number of introns per gene	5.9	5.3	4.4

Of the 40,884 transcripts identified, 84% were multi-exonic, with 5.9 exons each, on average, and longer introns than the previous gene annotations of Chandler (Table 2). The majority of intron/exon junctions were GT/AG-motif (98.2%), even though alternative splicing with non-canonical motifs was also observed (GC/AG: 0.8%; AT/AC: 0.11%). Almost 90% (36,422) of the coding sequences presented both canonical start and stop codons, while 4,462 had either a start or a stop codon. This result represents a great improvement compared with Chandler v1.0, where only 48% of the predicted gene models presented both start and stop codons [11].

Also, we observed that 2,801 gene models had from 2 to 4 transcript isoforms each, with a mean length of 9,389 bp. This proportion of gene models with multiple transcript isoforms is smaller than in other plant species [43, 44], likely owing to the low depth of coverage of our PacBio sequencing. Of the 6,437 isoforms identified, 1,448 were covered by FL HQ transcripts in ≥1 tissue, while 5,689 were expressed in ≥1 of the 20 tissues [11], which most likely covered higher gene diversity compared with the 9 tissues used for PacBio Iso-Seq. On average, the Illumina isoforms (9,188 bp) were longer than the PacBio isoforms (6,790 bp). By running the EnTAP functional annotation pipeline with the entire NCBI RefSeq plant database [45], we observed that almost all isoforms (98%; 6,287) were annotated with a plant protein.

We also investigated possible gene family expansion and contraction among the 3 Chandler versions' gene annotations. Overall, we identified fewer gene families in Chandler v2.0 (5,163 Panther family represented) than v1.0 (5,330) and NCBI RefSeq *J. regia* annotation (5,374). However, when counting the number of members per family, we observed a gene family expansion, in general, in Chandler v2.0 compared with v1.0: 39,357 proteins were assigned to a Panther gene family in Chandler v2.0, with an average of 7.6 members per family, against the 30,639 proteins annotated with a Panther domain in v1.0 (6 members per family on average). On the contrary, we noticed an overall gene family contraction in v2.0 compared to NCBI RefSeq, where 10.4 gene members were assigned to a Panther domain on average. Both the increment of contiguity and the reduction in haplotype redundancy can explain the observed patterns of gene family expansion and contraction among the 3 Chandler versions' gene annotations, even if the different methods of gene prediction used in the 3 studies could also account for these differences.

Most of the 1,440 core genes in the embryophyte dataset from BUSCO were assembled completely (82.5% single-copy; 12.6% duplicated), similarly to other *Fagales* genome assemblies (Table 3) [34, 35, 40, 46, 47]. Also, 88% of both rosids and green sets of core gene families (coreGFs) were identified in the gene annotation, confirming the HQ and completeness of the gene space of Chandler v2.0.

**Table 3:** Statistics of the completeness of Chandler v2.0 assessed with BUSCO and compared with other *Fagales* genomes

Genome	BUSCO complete (%)	BUSCO duplicated (%)	BUSCO fragmented (%)	BUSCO missing (%)	Reference
*Juglans regia*cv. “Chandler” v2.0	95.1	12.6	1.3	3.6	This genome
*Juglans regia*cv. “Chandler” v1.0	94.8	13.8	1.2	4.0	[11]
*Juglans regia*cv. “Serr” v1.0	94.5	11.1	1.5	4.0	[24]
*Fagus sylvatica* v1.2	94	19	1.7	3.6	[36]
*Castanea mollissima*	96.7	7.7	1.4	1.9	[35]
*Carya illinoinensis*v1	94	23	1.4	3.6	[34]
*Corylus avellane*cv. “Tombul”	96	6	1	3	[46]
*Quercus lobata* v1.0	90	52	4	6	[47]
*Quercus robur*	93	49	3	4	[36]

### Improved assessment of proteomes with the complete genome sequence

After confirming the importance of a chromosome-scale reference genome for the improvement of gene prediction accuracy, we analyzed the impact of a contiguous genome sequence using proteomic analysis. Proteomes are commonly investigated by isolating the total protein complement of a sample and fragmenting those proteins into smaller peptides that are resolved by mass and charge by means of mass spectrometry. After detection, the peptides’ amino acid sequences are determined by matching their mass and charge to candidate sequences obtained from a reference proteome inferred from the reference genome (virtual proteome). A fragmented assembly of the reference genome can lead to an inaccurate prediction of a species’ proteome and, then, a misidentification of the proteins expressed in specific tissues at particular stages [48].

We isolated proteins of reproductive tissues harvested from mature Chandler walnut trees, focusing on different development stages of the male flower (catkin; Supplementary Fig. S9) and mature pollen grains. We analyzed the proteomic data generated from these samples using the virtual proteomes predicted from the gene annotation of the new chromosome-scale genome and Chandler v1.0 (NCBI RefSeq). Considering all tissues analyzed, we identified fewer unique peptides (43,083) with the new chromosome-scale walnut genome than with Chandler v1.0 (44,679). In addition, 6,966 unique proteins were detected with Chandler v2.0 against the 8,802 found using version 1 as a search database (Supplementary Table S9; Additional File 3). Most likely, the NCBI proteomic database based on the fragmented Chandler v1.0 included artifacts resulting from an overestimation of the protein-coding genes.

**Figure 3:**
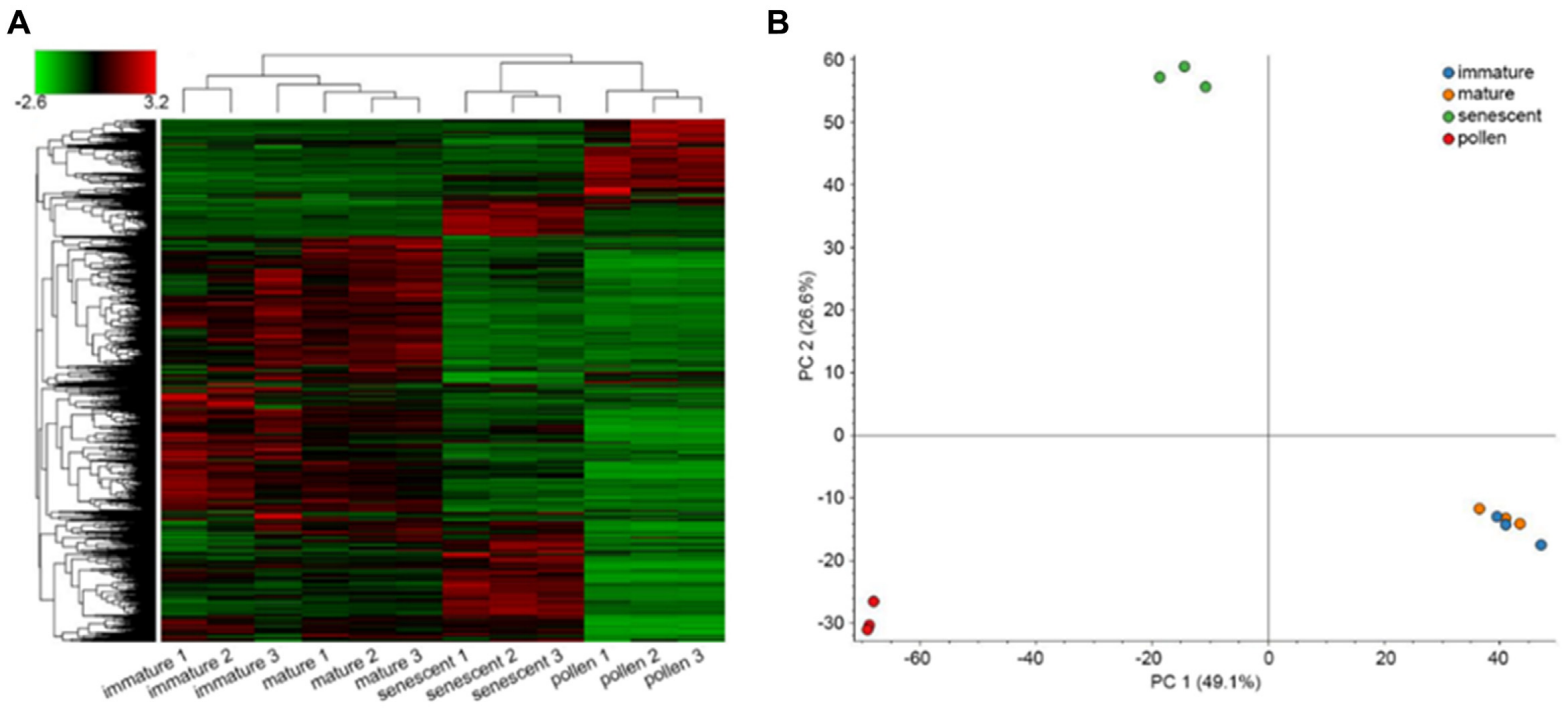
Clustering of the samples used in the proteomic analysis. (**A**) Hierarchical clustering based on Euclidian distances of normalized abundances of detected proteins. Samples are represented in columns and proteins in rows. (**B**) Principal component analysis of the 12 samples analyzed, clustering according to tissue type.

In the example presented below, we focused on the allergenic proteins produced during catkin and pollen development. Approximately 2% of walnut consumers have a high risk of developing allergies to nuts or pollen [49]. Initially, we clustered the samples according to their protein constituents and levels. This revealed a higher similarity between immature and mature catkins and a more distinct pattern of detected proteins between senescent catkins and pure pollen (Fig. 3).

We then searched the 4 analyzed proteomes for allergenic proteins listed in the World Health Organization/International Union of Immunological Societies Allergen Database (www.allergen.org; Supplementary Table S10), as well as for additional proteins not yet registered in the allergen database but predicted in Chandler v2.0 as potential allergens given their predicted structural similarity to known allergens (Additional File 3). Four of the 8 recognized allergenic proteins were detected in ≥1 of the catkin developmental stages, with Jug_r_5 (XP_018825777 | *Jr12_10750*) and Jug_r_7 (XP_018808763 | *Jr07_28960*) present in all sample types, including pollen (Supplementary Table S10). Genes adjacent to known allergen-coding sequences, likely indicating gene duplications, encode 3 of the new potential allergens (Supplementary Table S10). Moreover, we discovered that the gene locus *Jr12_05180* encodes a non-specific lipid transfer protein (Jug_r_9 | XP_018813928), a potential allergen highly expressed during catkin maturation and in pollen (Supplementary Tables S10 and S11). In particular, Jug_r_9 was the most abundant protein in mature and senescent catkins, and the second most abundant in pure pollen. Another interesting allergen similar to Jug_r_9 (same 8-cysteine configuration) is XP_018814382 | *Jr03_26970*; it decreases as the catkin matures and is absent in pollen (Supplementary Tables S10 and S11). Similarly, polyphenol oxidase (XP_018858848 | *Jr03_06780*) is high in the immature catkin and almost absent in the pollen.

The integration of these proteomic data with previously published transcriptomic data obtained from 20 walnut tissues [11] shows high reproducibility between the methods. In both datasets, allergens Jug_r_1, 4, and 6 were not detected in catkins, while the new putative allergen Jug_r_9 was highly expressed in catkins (Supplementary Tables S11 and S12). Also, *Jr12_05180* transcripts were not detected in any of the 20 tissues but catkin, thus confirming the strong specificity of Jug_r_9 for catkin and pollen tissue (Supplementary Table S12). Modeling the structure of this putative allergen reveals 4 predicted disulfide bonds, potentially conferring heat and protease resistance, and further suggesting allergenic properties (Supplementary Fig. S10). Future studies will clarify the functional role of this protein and its allergenic nature.

The detection of new potential walnut allergens confirms the positive impact of Chandler v2.0 on proteomic studies in walnut, by providing a clearer and more precise organization of the protein-coding sequences (CDSs) within a genomic region than the previous fragmented genome assembly v1.0.

### Chandler genomic diversity

By anchoring the HiRise assembly to the Chandler genetic map [16], we observed highly homozygous regions in Chandler, especially on Chr15, where the genetic gap spanned 14.5 cM, corresponding to a physical distance of 9.1 Mb. A large gap on Chr15 (9.23 cM; 1.5 Mb) was also observed by Luo et al. [37], which suggested inbreeding as a possible cause for the lack of segregating loci in this region in Chandler, whose parents shared Payne as an ancestor. To confirm the autozygosity of Chandler on Chr15, we used the Illumina whole-genome shotgun data of Chandler and the identified polymorphisms to study its genetic diversity across the new chromosome-scale genome. We identified 2,205,835 single heterozygous polymorphisms on the 16 chromosomal pseudomolecules, with an SNP density of 4.0 SNPs per kb (Fig. 2; Table S13). Fifty-six 1-Mb regions exhibited <377.5 SNPs (10th percentile of the genome-wide SNP number distribution), and chromosomes 15, 1, 7, and 13 were the top 4 chromosomes in the number of low-heterozygosity regions (Table S14). In particular, Chr15 presented 9 windows of 1 Mb with a significantly low number of polymorphisms, 5 of which span 4 Mb at the end of the chromosome. In these 9 low-heterozygosity regions, we found 1,536 SNPs in total (Fig. 2), of which only 25 were tiled on the Axiom *J. regia* 700 K SNPs array. The absence of these polymorphisms segregating in Chandler in the SNP array could be related to either a failed identification during the SNP calling due to the highly fragmented reference genome v1.0 or to the SNP exclusion during the filtering process applied to build the genotyping array [9]. The low number of Chandler heterozygous SNPs in the array affected the end of Chr15 the most, causing a reduction in the genetic length of the corresponding LG (Fig. 1), as well as leaving unexplored 4 Mb of Chandler genetic variability, which is now accessible thanks to the new chromosome-scale reference genome. The failure to anchor 7 of the scaffolds with telomeric sequences can be explained by the missed detection of terminally located highly homozygous regions during genetic map constructions, due to the absence of crossing-over events with heterozygous flanking markers.

Owing to the evidence of whole-genome duplication in *Juglans* genomes [37], we searched for conserved regions of synteny between Chr15 and its homologous regions in the genome, to study their level of divergence and identify other evolutionary forces as possible causes of the localized reduction of heterozygosity on Chr15. Of the 5,739 pairs of paralogous genes (8,701 genes; Supplementary Fig. S11) identified in Chandler v2.0, 448 included genes on Chr15, and 389 of these have their respective paralogues on Chr6 (Supplementary Fig. S12), in line with what was already reported by Luo et al. [37]. The Chr06-Chr15 pairs of paralogous genes showed average values of divergence indexes (*K_S_* = 0.38; *K_A_* = 0.13) similar to the ones observed genome-wide for other syntelogs (*K_S_* = 0.40; *K_A_* = 0.09), which are paralogous genes derived from the same ancestral genomic region. Similar values of divergence were also observed for the 178 Chr06-Chr15 syntelogs (171 genes) falling within the 9 low-heterozygosity regions on Chr15 (*K_S_* = 0.40, *K_A_* = 0.10), excluding different evolutionary rates for these regions. Other than paralogous genes, we found 393 singleton genes in the low-heterozygosity regions on Chr15 of Chandler. These genes are involved in different biological processes, many of which are related to signal transduction, protein phosphorylation, and response to environmental stimuli (Supplemenary Table S15).

**Figure 4:**
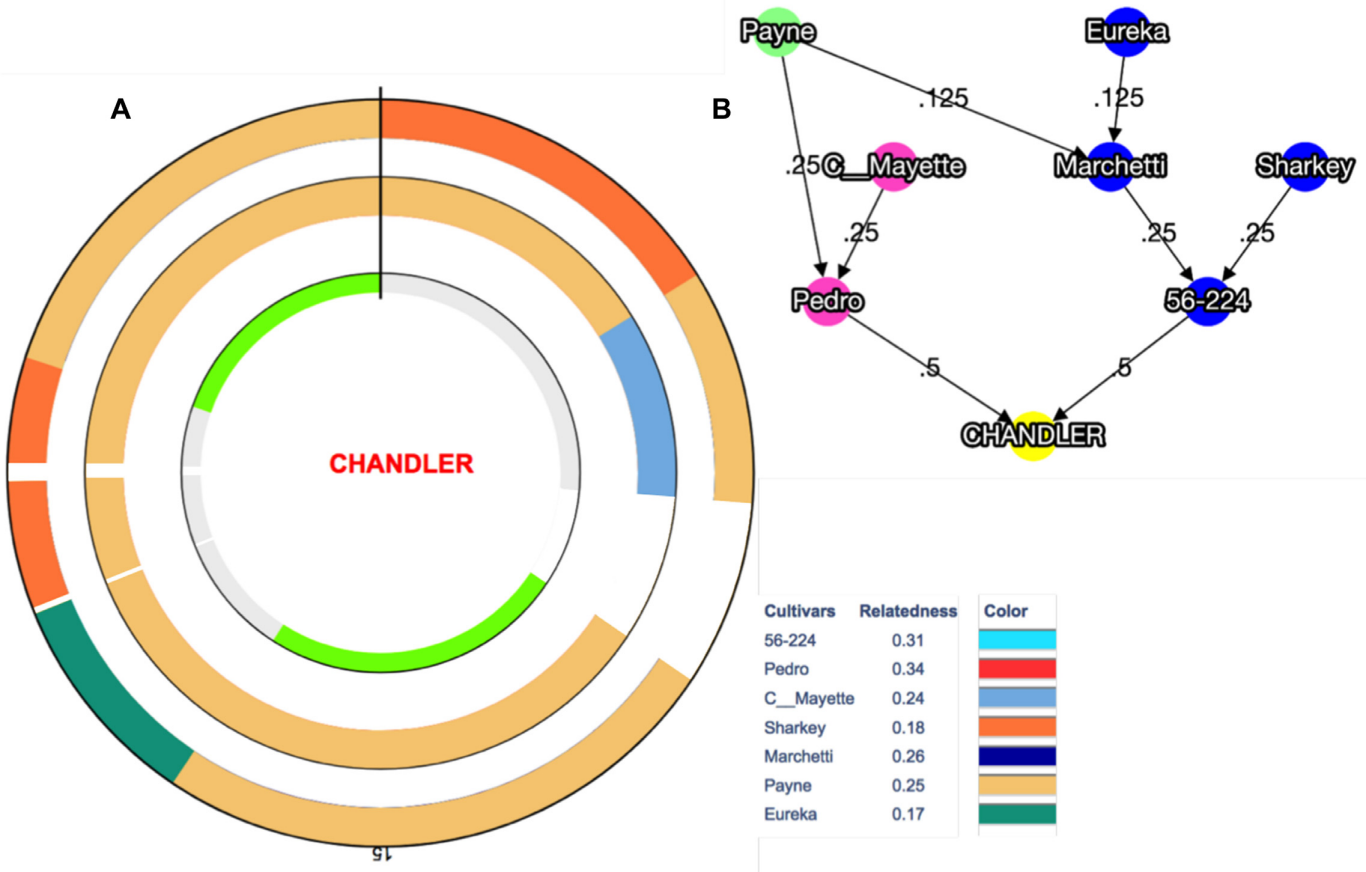
Graphical visualization of haplotype block (HB) inheritance on Chr15 along with the Chandler pedigree. (**A**) The inner circle highlights in grey 2 regions of heterozygosity (5 HB the first and 7 HB the second), and in light green 2 regions of homozygosity (3 HB the first and 4 HB the second). The circle in the middle shows maternally inherited HBs, while the HBs inherited through the paternal line are visualized in the outer circle. Payne's haplotypes are clearly present in both parental lines. White spaces represent segments of missing haplotype information. (**B**) Chandler pedigree, where Pedro is the maternal line and 56–224, the paternal line.

**Figure 5:**
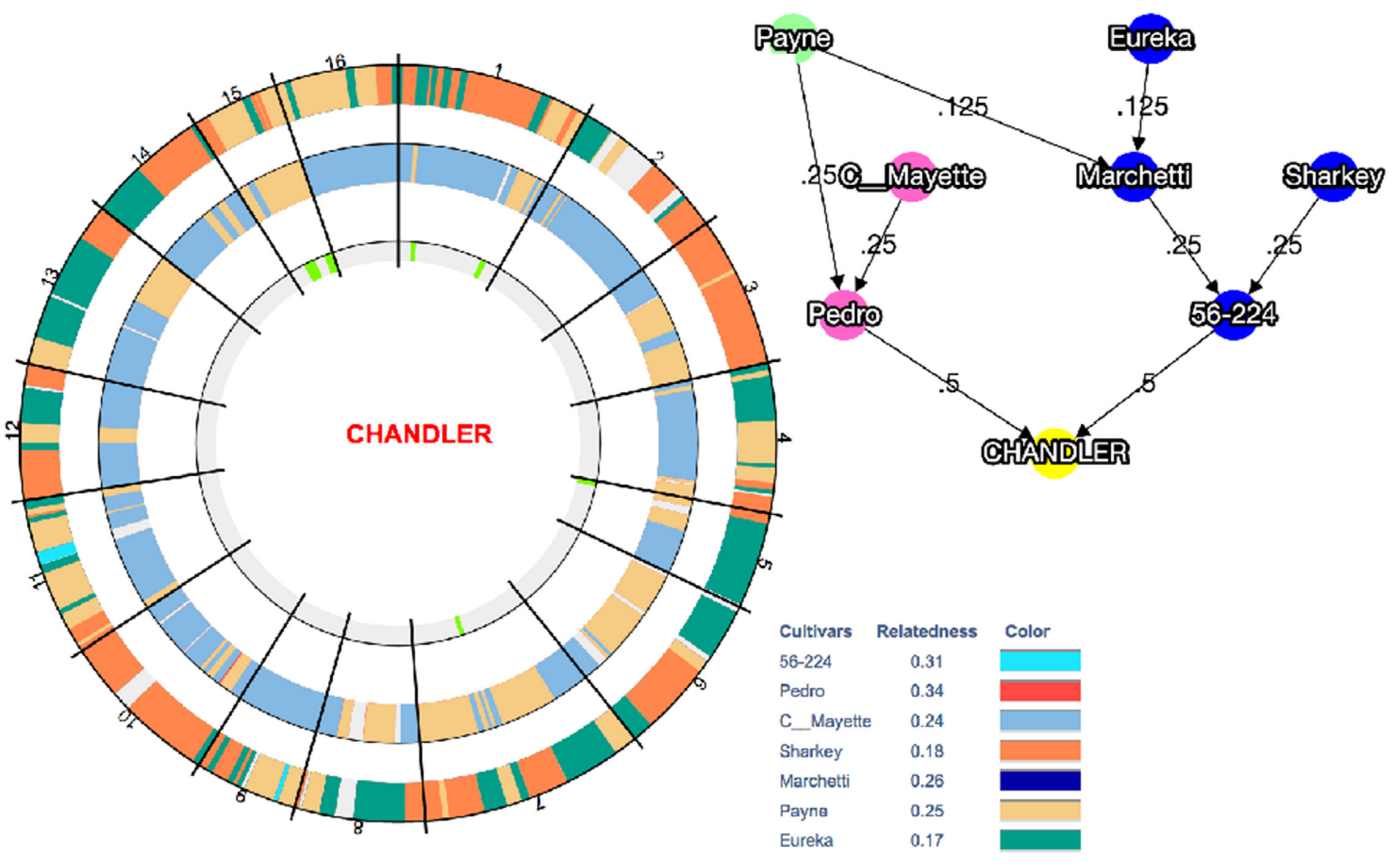
Graphical visualization of the haplotype block (HB) inheritance across Chandler pedigree in the 16 chromosomes. The inner circle highlights in grey the regions of heterozygosity and in light green the regions of homozygosity for each chromosome. The circle in the middle shows the maternally inherited HBs, while the HBs inherited from the paternal line are visualized in the outer circle. In both parental line circles, missing data are highlighted in grey. Payne haplotypes are inherited along both parental lines in all chromosomes but Chr5, Chr9, Chr10, Chr14, and Chr16. Chandler pedigree is represented on the side, where Pedro is the maternal line and 56–224 the paternal line.

We further investigated the contribution of inbreeding to the high level of autozygosity on Chr15 by visualizing the inheritance of haplotype blocks (HB; genomic regions with little recombination) across the Chandler pedigree (Fig. 4B). We observed that Payne accounts for the entire Chandler genetic makeup (19 HBs for the total length of Chr15) inherited from Pedro (mother), where only 1 HB (2.08 Mb) shared the same allele of Conway-Mayette (maternal grandfather; Fig. 4A). Regarding the paternal genetic makeup of Chandler, 13 of 19 HBs (9.05 Mb) on Chr15 inherited Payne alleles, providing further evidence of high inbreeding on this chromosome (Fig. 4A). This is even more evident in assessing the number of alleles matching between Payne and Chandler across the genome: Chr15 (14 HBs for a total of 13.95 Mb; Supplementary Fig. S13) shares full allele identity with Payne for almost its entire length. Such allele matching between Chandler and its ancestor Payne also occurs on Chr1 (9 HBs for a total of 8.44 Mb), Chr4 (6 HBs; 7.68 Mb), Chr7 (21 HBs; 21.62 Mb), and Chr14 (7 HBs; 12.29 Mb; Fig. 5). These results suggest a high level of inbreeding in many genomic regions of Chandler (Fig. 5), even though direct and indirect selection might have caused the observed presence of extended homozygous regions in Chandler's genome.

### Genomic comparison between Eastern and Western walnuts

Even though numerous surveys regarding genetic diversity within walnut germplasm collections have been reported so far [50, 51], comparative analyses at the population level and genome scans for signatures of selection are still missing in Persian walnut. The availability of a chromosome-scale reference genome enables exploration of the patterns of intraspecific variation at the genomic level, providing new insight on the extraordinary phenotypic diversity present within *J. regia*.

We used the resequencing data generated for 23 founders of the Walnut Improvement Program of the University of California, Davis (UCD-WIP; Supplementary Table S16) [12], to study the genome-wide genetic differentiation among walnut genotypes of different geographical provenance. We identified 14,988,422 SNPs, and >97% of them were distributed on the 16 chromosomal pseudomolecules, with 9.4 polymorphisms per kilobase pair. A hierarchical clustering analysis (Supplementary Fig. S14) divided the 23 founders into 2 major groups, including genotypes from Western countries (USA, France, and Bulgaria) and Asia (China, Japan, Afghanistan), respectively, as previously reported [9, 52]. High phenotypic diversity for many traits of interest in walnut, such as phenology, nut quality, and yield, has been observed within and between germplasm collections from Western and Eastern countries [53]. Walnut trees from Asia are noted for their lateral fruitfulness and precocity, rarely observed in the USA and western Europe, so they have been used as a source of these phenotypes in different walnut breeding programs [54].

At a genomic level, we found a moderate differentiation (*F_ST_* = 0.15) between Western and Eastern genotypes, except for 195 genomic windows (100 kb) that showed substantially high population differences (*F*_ST_ ≥ 0.36; top 5% in the whole genome). In particular, chromosomes 7, 5, 1, 4, and 2 presented ∼70% of the divergent sites (Fig. 2; Supplementary Fig. S15). As suggested by the mean reduction of diversity coefficient (ROD) value (0.41), in most of the genomic regions highly differentiated, the UCD-WIP founders from the USA and Europe showed lower nucleotide diversity (π = 2.5 × 10^−4^) than the Asian genotypes (π = 5.0 × 10^−4^), consistent with Bernard et al. [10] (Fig. 2; Supplementary Fig. S15). The proximity of our eastern genotypes to the supposed walnut center of domestication in central Asia can explain the high level of diversity observed in this subgroup.

More than 60% (122) of the highly differentiated windows showed a negative value of Tajima D in the EU/USA subgroup (*D*_Occ_ = −1.12), thus suggesting that selection has been likely acting on these genomic regions in the Western genotypes (Supplementary Fig. S15). Here we found 743 genes, with GO biological categories mostly related to signal transduction, embryo development, and response to stresses (Table S17). Ten candidate selective sweeps (*D*_Asia_ = −0.54) were also observed in the Eastern group (Supplementary Fig. S15), which included 57 predicted genes, related to terpenoid biosynthesis, post-embryonic development, and signal transduction (Supplementary Table S18).

Recently, many marker-trait associations have been reported for different traits of interest in walnut, such as leafing date, nut-related phenotypes, and water use efficiency [14–16]. We looked to see whether any of these trait-associated SNPs fell within regions highly differentiated between Western and Eastern genotypes. Three loci associated with shape index, nut roundness, and nut shape [14] are located in 2 genomic regions on chromosome 3 and 4 with significantly high values of *F*_ST_ (Supplementary Table S19). In both of these regions, Western genotypes presented lower genetic diversity and lower values of Tajima D than the Eastern walnuts. These findings may suggest that, while a selective pressure for nut shape may have occurred in the EU/USA subgroups, higher phenotypic variability can be expected for these traits in the Eastern countries. We also found that the locus AX-170770379, strongly associated with harvesting date [16], falls within a genomic region on Chr1 with an *F*_ST_ value equal to 0.39 and lower genetic diversity in the Western genotypes (ROD = 0.63; Supplementary Table S19). Looking at the phenotypic effect of this SNP on the harvest date of the 23 founders, we observed that most of the Western genotypes are later harvesting than the Eastern (Supplementary Fig. S16), suggesting differences in the timing of phenological events between these 2 groups as adaptation to the different climate conditions present in their countries of origin [55].

Future resequencing projects involving larger walnut collections and covering a wider area of the global walnut distribution are necessary to confirm and interpret the observed genomic differentiation between Western and Eastern walnuts, likely helping to elucidate the role of this genomic divergence in the evolutionary history of Persian walnut.

## Methods

### Oxford Nanopore sequencing and assembly

High molecular weight (HMW) DNA for Nanopore sequencing (Oxford Nanopore Technologies Inc., UK) was isolated through a nucleus extraction and lysis protocol. First, mature leaf tissue from the same tree used for the original *J. regia* Chandler genome [11] was homogenized with mortar and pestle in liquid nitrogen until well ground, then added to the Nuclei Isolation Buffer [56] and stirred at 4°C for 10 minutes. The cellular homogenate was filtered through 5 layers of Miracloth (Millipore-Sigma) into a 50-mL Falcon tube, then centrifuged at 4°C for 20 minutes at 3,000*g*. This speed of centrifugation was selected on the basis of the estimated walnut genome size of 1 Gb [57]. Extracted nuclei were then lysed for 30 minutes at 65°C in the sodium dodecyl sulfate–based lysis buffer described by Mayjonade et al. [58]. Afterwards, 0.3 volumes of 5 M potassium acetate were added to the lysate to precipitate residual polysaccharides and proteins. The sample was incubated for 5 minutes at 4°C and then centrifuged at 4°C for 10 minutes at 2,400*g*. After removal of the supernatant, genomic DNA (gDNA) was ethanol precipitated and then eluted in 10 mM Tris-Cl. Further purification of the gDNA was then performed using a Zymo Genomic DNA Clean and Concentrate column.

A 1-µg aliquot of the isolated gDNA was prepared for sequencing using the Ligation sequencing kit (LSK108, ONT) following the manufacturer's protocol with an optimized end repair (100 µL sample, 14 µL enzyme, 6 µL enzyme, incubated at 20°C for 20 minutes then 65°C for 20 minutes). In detail, the gDNA was end polished using the NEBNext® Ultra™ II DNA Library Prep Kit and then cleaned up with 1X Ampure XP beads (Beckman Coulter). Afterwards, the gDNA was ligated to ONT-specific adapters, followed by an additional cleanup with 0.4X Ampure XP beads. Finally, the libraries were sequenced for 48 hours on 6 flowcells of the ONT Mk1B MinION platform with the R9.4 chemistry. Raw fast5 data were base-called using Albacore version 1.25.

The ONT data and Illumina reads from Martínez-García [11] were combined to obtain the Chandler hybrid assembly using MaSuRCA v3.2.3 [59]. In detail, MaSuRCA first transformed the Illumina paired-end reads into “super-reads” using the super-reads algorithm, which uses *k*-mers from Illumina reads to extend each Illumina read uniquely in both directions. Then, each ONT read was used as a template to which super-reads can be attached, and the approximate alignments of all super-reads to each ONT read were computed. The best path of the exactly overlapping aligned super-reads on an ONT read was then defined, generating a “mega-read.” The mega-reads typically have a very low error rate (<1%) because they are constructed from the super-reads, and most of them span the full length of the long reads. Finally, a customized version of the CABOG assembler [60] was used to assemble the mega-reads along with the Illumina mate pairs, which provide the linking information for the scaffolding. Gaps were closed using the gap-filling procedure implemented in MaSuRCA and described by Zimin et al. [59]. The de-duplication module implemented in MaSuRCA was then applied to remove duplicative sequences (scaffold variants due to heterozygosity).

De-duplicated scaffolds were aligned onto the previously finished *J. regia* chloroplast genome [11] using “minimap2 -x asm5,” as well as to a database of 223 finished plant mitochondria (downloaded from NCBI RefSeq) using blastn with default parameters. Finally, Chandler v1.0 was aligned to the de-duplicated hybrid assembly, and the unaligned regions were added to the Chandler hybrid assembly.

### Hi-C sequencing

A Hi-C library was prepared by Dovetail Genomics LLC (Santa Cruz, CA, USA) as described previously [61]. Briefly, for each library, chromatin was fixed in place with formaldehyde in the nucleus and then extracted. Fixed chromatin was digested with DpnII, the 5′ overhangs filled in with biotinylated nucleotides, and then free blunt ends were ligated. After ligation, crosslinks were reversed and the DNA purified from protein. Biotin that was not internal to ligated fragments was removed from the purified DNA. Purified DNA was then sheared to ∼350 bp mean fragment size. Sequencing libraries were generated using NEBNext^®^ Ultra^TM^ enzymes and Illumina-compatible adapters. Biotin-containing fragments were isolated using streptavidin beads before PCR enrichment of each library. The libraries were then sequenced on the Illumina HiSeq4000 platform.

The hybrid ONT assembly, Illumina shotgun reads [11], and Dovetail Hi-C library reads were used as input data for the scaffolding software HiRise, which uses proximity ligation data to scaffold genome assemblies [62]. Shotgun and Dovetail Hi-C library sequences were aligned to the hybrid ONT assembly using a modified SNAP read mapper. The separations of Dovetail Hi-C read pairs mapped within the ONT scaffolds were analyzed by HiRise to produce a likelihood model for the genomic distance between read pairs, and the model was used to identify and break putative misjoins, to score prospective joins, and make joins above a threshold. After scaffolding, Illumina shotgun sequences were used to close gaps between contigs, resulting in an improved HiRise assembly.

### Validation and anchoring of the HiRise assembly to Chandler genetic maps

The HiRise assembly was first anchored to the Chandler genetic map obtained by Marrano et al. [16] from a 312-offspring F_1_ population “Chandler × Idaho” genotyped with the latest Axiom *J. regia* 700 K SNP array. SNP probes (71-mers including the SNP site) from the Axiom *J. regia* 700 K SNP array were aligned onto the HiRise assembly, filtering out alignments with probe/reference identity <98%, covering <95% of the probe length, or aligning multiple times on the genome. Retained markers with a unique segregation profile were then used to anchor the HiRise scaffolds. The same procedure was also followed to anchor the HiRise assembly to the Chandler genetic map used to construct a walnut bacterial artificial chromosome clone-based physical map by Luo et al. [37]. The final ordering of scaffolds was performed by taking into consideration the marker genetic map position, and, in the final sequence, consecutive scaffolds were separated by sequences of 100,000 nucleotides.

The Tandem Repeat Finder program (TRF v4.09 [63]) was run using the recommended parameters (max mismatch delta PM PI minscore maxperiod, 2 7 7 80 10 50 500, respectively) to identify repeat elements up to 500 bp long. A histogram of repeat unit lengths was generated, and peaks at 7, 29, 33, 44, 154, and 308 bp were identified. From these data, a consensus sequence corresponding to each peak was selected. All of these repeat sequences were aligned onto the HiRise assembly using “nucmer” from the MUMmer4 package [64] with a minimum match length of 7 to capture the telomeric repeat. On the basis of the positions of these alignments along the chromosomes and contigs, we identified the 7-mer as the telomeric repeat and the 154-mer and 308-mer as centromeric repeats.

Recombination rate was estimated within sliding windows of 10 Mb with a step of 1 Mb along the chromosome sequence by using the high-density genetic map of Chandler [16] and the R/MareyMap package v1.3.4 [65]. To evaluate the Chandler v2.0 error rate, the 2 assemblies, Chandler v1.0 and 2.0, were aligned to each other using nucmer [64]. Assembly quality statistics were estimated using QUAST v5.0.2 [66], filtering for contigs with a minimum length of 1 kb. The haploid size of the walnut genome was estimated by first generating the 24-mer distribution of Illumina paired-end reads (54-fold coverage of the haploid genome) with Jellyfish v2.2.6 [67] and then uploading it to Genomescope [68]. Comparisons of Chandler v2.0 versus JrSerr_v1.0 and vice versa were performed using nucmer [64], and then the function “dnadiff” implemented in MUMmer4 was used to obtain detailed information on the differences between 2 assemblies.

### RNA preparation

Five walnut tissues (leaf, catkin 1-inch elongated, catkin 3-inches elongated, pistillate flower, and pollen) were collected from Chandler trees at the UCD walnut orchards. Four additional samples (somatic embryo, callus, shoot, and roots) were taken from tissue culture material of Chandler. Several grams of each tissue were ground in liquid nitrogen and with insoluble polyvinylpyrrolidone (1% w/w). RNA was isolated using the PureLink™ Plant RNA Reagent (Invitrogen^TM^, Carlsbad, CA) following the manufacturer's instructions, but with an additional end wash in 1 mL of 75% ethanol. For root tissue only, RNA isolation was performed using the MagMAX^TM^ mirVana^TM^ Total RNA Isolation Kit (Applied Biosystems^TM^, Foster City, CA) as per protocol, except for the lysis step. A different lysis buffer was created adding 100 mg of sodium metabisulfite to 10 mL of guanidine buffer (guanidine thiocyanate 4 M, sodium acetate 0.2 M, EDTA 25 mM, PVP-40 2.5%, pH 5.0) and 1 mL of nuclease-free water. Then, 100 mg of ground root tissue was lysed in 1 mL of the new lysis buffer using a Tissue Lyser at maximum frequency for 2 minutes. The lysate was centrifuged at 4°C for 5 minutes at maximum speed. The supernatant (500 µL) was transferred to a new tube for the following steps of RNA isolation as per protocol. RNA samples were then purified, and DNase treated using the RNeasy Plant Mini Kit (Qiagen, Hilden, Germany). The RNA quality was confirmed by running an aliquot of each sample on an Experion^TM^ Automated Electrophoresis System (Bio-Rad, Hercules, CA).

### PacBio Iso-Seq sequencing

Full-length cDNA Iso-Seq template libraries for PacBio Iso-Seq analysis were constructed and sequenced at the DNA Technologies & Expression Analysis Core Facility of the UCD Genome Center. FL double-stranded cDNA was generated from total RNA (2 µg per tissue) using the Lexogen Telo^TM^ prime Full-length cDNA Kit (Lexogen, Inc., Greenland, NH). Tissue-specific cDNAs were first barcoded by PCR (16–19 cycles) using IDT barcoded primers (Integrated DNA Technologies, Inc., Coralville, IA), and then bead-size selected with AMPure PB beads (2 different size fractions of 1X and 0.4X). The 9 cDNAs were pooled in equimolar ratios and used to prepare a SMRTbell™ library using the PacBio Template Prep Kit (PacBio, Menlo Park, CA). The SMRTbell™ library was then sequenced across 4 Sequel v2 SMRT cells with polymerase 2.1 and chemistry 2.1 (P2.1C2.1).

PacBio raw reads were processed using the Iso-Seq3 v.3.0 workflow following PacBio recommendations [69]. CCSs were generated using the program “ccs.” The CCSs were demultiplexed and cleaned of cDNA primers using the program “lima.” Afterward, CCS clustering and polishing was performed using the program “Iso-Seq3,” to generate HQ FL sequences for each of the 9 tissues. FLnc and HQ clusters were aligned onto the new “Chandler” assembly v2.0 with minimap2 v.2.12-r827, including the parameter “-ax splice” [70].

### Repeat annotation

A genome-specific repeat database was created using the “basic” mode implemented in RepeatModeler v.1.0.11 [71]. RepeatMasker v.4.0.7 was then run to mask repeats in the walnut reference genome v.2.0 and generate a GFF file [72].

### Gene prediction and functional annotation

*Juglans regia* RefSeq transcripts and additional *J. regia* transcripts and protein sequences downloaded from NCBI, along with the HQ FL Iso-Seq transcripts, were used as input to the PASA pipeline v.2.3.3 [73], to assemble a genome-based transcript annotation. PASA ushe aligners BLAT v.35 [74] and GMAP v.2018–07-04 [75], along with TransDecoder v.5.5.0 [76], which predicts open reading frames as genome-based GFF coordinates. The final PASA/TransDecoder GFF3 file was post-processed to name the genes and transcripts by chromosome location consistently. The chloroplast and mitochondrial genomes were annotated using the “CHLOROBOX GeSeq Annotation of Organellar Genomes” tool at [77] with default parameters [78]. NCBI accessions NC_028617.1 (*J. regia* chloroplast), KT971339.1 (*Medicago truncatula* mitochondrion), NC_029641.1 (*M. truncatula* mitochondrion), and NC_012119.1 (*Vitis vinifera* mitochondrion) were also input as custom references. The output gff3 files were then post-processed to consistently rename genes.

Functional roles were assigned to predicted peptides using Trinotate v.3.1.1 [79]. In particular, similarity searches were performed against several public databases (i.e., Uniprot/Swiss-Prot, NCBI NR, *Vitis_vinifera.IGGP_12x, J. regia* RefSeq) using BLAST v.2.8.1, HMMER v.3.1b2, SignalP v.4.1c, and TMHMM v.2.0c. Gene family analysis was performed by running Interproscan v. 5.30–69.0 [80, 81] with default parameters on each protein fasta file (v1.0 [11], NCBI RefSeq [GCF_0 014 11555.1_wgs.5d] and v2.0). The PANTHER family ID with the lowest expect value (below expect value threshold of 1.0E^−11^) was assigned to each protein.

The completeness and quality of both genome assembly and gene annotation of Chandler v.2.0 were estimated with the BUSCO method v.3 (1,440 core genes in the embryophyte dataset) [82], and the sets of coreGFs of green plants (2,928 coreGFs) and rosids (6,092 coreGFs) from PLAZA v.2.5 [83]. Also, RNA-sequencing (RNA-Seq) data previously generated for 20 tissues (see [11]) were aligned to the reference genome (v1.0 and v2.0) with HISAT2 [84]. The alignments of the 20-tissue RNA-Seq data and the FL transcripts along with the new genome annotation v2.0 were then used as input to StringTie v.2.0 [85] to estimate expression levels in both fragments per kilobase per million reads (FPKM) and transcripts per million (TPM) for each transcript in the v2 annotation. The percent identity and coverage of each *J. regia* transcript compared to proteins in the NCBI plant RefSeq database was also determined by running the EnTAP pipeline v.0.9.0 [45].

### Label-free shotgun proteomics

Plant tissues of immature, intermediate, and mature catkins (Additional File 1, Supplementary Fig. S9) and pure pollen from 3 individual trees of Chandler at the UCD walnut orchards were collected and frozen immediately in dry ice. Tissues were then further frozen in liquid nitrogen in the laboratory and ground with mortar and pestle. Five hundred milligrams of each sample were used for total protein extraction, following the procedure for recalcitrant plant tissues of [86], with a modification in the final buffer used to resuspend the protein pellet, consisting of 8 M urea in 50 mM triethylammonium bicarbonate (TEAB). Then, 100 μg of total protein from each sample was used for proteomics.

Initially, 5 mM dithiothreitol (DTT) was added and incubated at 37°C for 30 minutes and 1,000 rpm shaking. Next, 15 mM iodoacetamide (IAA) was added, followed by incubation at room temperature for 30 minutes. The IAA was then neutralized with 30 mM DTT in incubation for 10 minutes. Lys-C/trypsin then was added (1:25 enzyme: total protein) followed by 4 h incubation at 37°C. Afterward, TEAB (550 μL of 50 mM) was added to dilute the urea and activate trypsin digestion overnight. The digested peptides were desalted with Aspire RP30 Desalting Tips (Thermo Scientific), vacuum dried, and suspended in 45 μL of 50 mM TEAB. Peptides were quantified by Pierce quantitative fluorometric assay (Thermo Scientific) and 1 μg analyzed on a QExactive mass spectrometer (Thermo Scientific) coupled with an Easy-LC source (Thermo Scientific) and a nanospray ionization source. The peptides were loaded onto a Trap (100 μm, C18 100 Å 5 U) and desalted online before separation using a reversed-phase (75 μm, C18 200 Å 3 U) column. The duration of the peptide separation gradient was 60 minutes using 0.1% formic acid and 100% acetonitrile for solvents A and B, respectively. The data were acquired using a data-dependent tandem mass spectroscopy (MS/MS) method, which had a full scan range of 300–1,600 Da and a resolution of 70,000. The resolution of the MS/MS method was 17,500 and the insulation width 2* m*/*z* with a normalized collision energy of 27. The nanospray source was operated using a spray voltage of 2.2 kV and a transfer capillary temperature heated to 250°C. Samples were analyzed at the UCD Proteomics Core.

The raw data were analyzed using X! Tandem and viewed using the Scaffold Software v.4 (Proteome Software, Inc.). Samples were searched against UniProt databases appended with the cRAP database, which recognizes common laboratory contaminants. Reverse decoy databases were also applied to the database before the X! Tandem searches. The CDSs annotated in Chandler v1.0 (NCBI accession PRJNA350852) and v2.0 were used as a reference for identification of proteins from the mass spectrometry data. The proteins identified were filtered in the Scaffold software based on the following criteria: 1.0% FDR (false-discovery rate) at protein level (following the prophet algorithm [87]), the minimum number of 2 peptides, and 0.1% FDR at the peptide level. Structure of the walnut allergen (Jug r 9) was modelled using SWISS-MODEL [88] based on the structure of a homologous allergen from lentil (PDBid:2MAL). Structures were superimposed using MUSTANG (2MAL: in red, walnut in blue) [89].

### Chandler genomic diversity

Illumina whole-genome shotgun data of Chandler were aligned on Chandler v2.0 with BWA [90] with standard parameters. SNP calling was performed using SAMtools v1.9 [91] and BCFtools v.2.1 [92]. SNP density for windows of 1 Mb was estimated using the command “SNPdensity” implemented in VCFtools v0.1.16 [93]. Self-collinearity analysis to detect duplicated regions in Chandler v2.0 was performed with MCScanX [94], using a simplified GFF file of the new gene annotation and a self-BLASTP as input. To improve the power of collinearity detection, tandem duplications were excluded after running the function “detect_collinear_tandem_arrays” implemented in MCScanX. Synonymous (*K_S_*) and nonsynonymous (*K_A_*) changes for syntenic protein-coding gene pairs were measured using the Perl script “add_ka_and_ks_to_collinearity.pl” implemented in MCScanX.

To explore the inbreeding level across the 16 chromosomal pseudomolecules of Chandler, haplotypes were built for 55 individuals of the UCD-WIP, including 25 founders and several commercially relevant walnut cultivars (e.g., Chandler, Howard, Tulare, Vina, Franquette) along with their parents and progenitors. All individuals were genotyped using the latest Axiom^TM^*J. regia* 700 K SNP array as described in [9]. To define SNP HBs, 26,544 unique and robust SNPs were selected and ordered according to the Chandler genome v2.0 physical map. Subsequently, for each SNP marker and individual, phasing and identification of closely linked groups of SNPs, without recombination in most of the pedigree, was performed using the software FlexQTL^TM^ [95] and PediHaplotyper [96] following the approach described in [96] and [97]. In particular, HBs were defined by recombination sites detected in ancestral generation of Chandler.

### Genomic comparison between Eastern and Western walnuts

The resequencing data of 23 founders of the UCD-WIP (Table S16) [12] were mapped onto the Chandler v2.0 with BWA, and SNPs were called following the same procedure described above for Chandler. SNPs with no missing data and minor-allele frequency >10% were retained for the following genetic analyses (7,269,224 SNPs out of the 14,988,422 identified). Hierarchical cluster analysis on a dissimilarity matrix of the 23 UCD-WIP founders was performed using R/SNPRelate v.1.18.0 [98]. Fixation index (*F*_ST_) was measured between genotypes from EU/USA and Asia with VCFtools v0.1.16, setting windows of 100 and 500 kb. Genomic windows with the top 5% of *F*_ST_ values were selected as candidate regions for further analysis. The empirical cut-off with a low FDR (5%) was verified by performing whole-genome permutation test (1,000) with a custom Python script. Nucleotide diversity (π) and Tajima D [99] were also computed along the whole genome in 100- and 500-kb windows using VCFtools. Reduction of diversity coefficient was estimated as 1 – (π _Occ_/π_Asia_). The new walnut gene annotation v.2.0 was used to identify predicted genes in the candidate regions under selection. The distribution of the identified genes into different biological processes was evaluated using the weight01 method provided by R/topGO [100]. The Kolmogorov-Smirnov–like test was performed to assess the significance of over-representation of GO categories compared with all genes in the walnut gene prediction. Plots were obtained using the R/circlize v.0.4.6 and R/ggplot2 v.3.5.3 packages.

## Availability of Supporting Data and Materials

All raw and processed sequencing data generated in this study have been submitted to the NCBI BioProject database [101] under accession number PRJNA291087. All SNP data have been submitted to Hardwood Genomics [102]. Data further supporting this work are openly available in the *GigaScience* repository, GigaDB [103].

## Additional Files

**Supplementary Figure S**1. Contiguity of the Chandler ON assembly and the final HiRise scaffolds.

**Supplementary Figure S**2. Mapping positions of the first and second read in the read pair respectively, grouped into bins.

**Supplementary Figure S**3. Collinearity between the ‘Chandler’ genetic map of [35] and the 16 chromosomal pseudomolecules of Chandler v2.0.

**Supplementary Figure S**4. Whole-genome comparison between Chandler v2.0 and JrSerr_v1.0 [22].

**Supplementary Figure S**5. Retrotransposons distribution across the 16 chromosomes of Chandler v2.0.

**Supplementary Figure S**6. Top 20 biological process GO terms.

**Supplementary Figure S**7. Top 20 molecular function GO terms.

**Supplementary Figure S**8. Top 20 cellular component GO terms.

**Supplementary Figure S**9. Catkin development stages sampled for the label-shotgun proteomic study (pollen is not shown).

**Supplementary Figure S**10. Modeled structure of the putative new allergen encoded by *Jr12_05180*.

**Supplementary Figure S**11. Collinear blocks among the 16 chromosomal pseudomolecules of Chandler v2.0.

**Supplementary Figure S**12. Dual synteny plot between Chr06 a- Chr15 of Chandler v2.0.

**Supplementary Figure S**13. Graphical visualization of allele identity between Chandler and its ancestor Payne for all 16 chromosomes of Chandler.

**Supplementary Figure S**14. Hierarchical clustering analysis among the 23 re-sequenced founders of the UCD-WIP.

**Supplementary Figure S**15. Genome scan for selective sweeps between walnuts from EU/USA and Asia.

**Supplementary Figure S**16. Phenotypic differences of harvesting date observed among the three genotypic classes of the marker AX-170770379 significantly associated to harvest date (Marrano et al., 2019).

Supplementary Table 1. Statistics on the six Nanopore flowcells used to sequence Chandler’s genome.

Supplementary Table 2. Statistics on k-unitigs, super-reads and mega-reads obtained with the MaSuRCA assembler on ONT and Illumina reads.

Supplementary Table 3. Characteristics of the Chandler ONT assembly.

Supplementary Table 4. List of tissues used for PacBio IsoSeq.

Supplementary Table 5. Statistics on the PacBio IsoSeq sequencing per flow-cell.

Supplementary Table 6. Statistics on CCSs, FLnc and HQ FL transcripts obtained per tissue with PacBio IsoSeq.

Supplementary Table 7. Percentage of FLnc and HQ FL transcripts aligned on the new assembly per tissue.

Supplementary Table 8. Validation of gene annotation with expression data and public plant protein databases.

Supplementary Table 9. Summary of proteome results obtained using genome annotations v1 and v2.

Supplementary Table 10. Allergen expression data obtained from proteome data.

Supplementary Table 11. Top 10 abudant proteins detected in each tissue.

Supplementary Table 12. Expression of genes encoding allergenic proteins in different *Juglans regia* cv. Chandler tissues.

Supplementary Table 13. Number of SNPs and SNP density in 100-kb windows per chromosome in Chandler v2.0.

Supplementary Table 14. Regions (1 Mb) with less than 377.5 SNPs (10th percentile of the SNP number distribution) in Chandler.

Supplementary Table 15. Top 50 biological process GO terms for the 393 singletons genes in the low heterozygous regions on Chr15 of Chandler.

Supplementary Table 16. List of 23 UCD-WIP founders used for the selective sweep analysis .

Supplementary Table 17. Top 50 biological process GO terms for the 122 windows (100 kb) with negative value of Tajima’s D in the Western genotypes.

Supplementary Table 18. Top 50 biological process GO terms for the 122 windows (100 kb) with negative value of Tajima’s D in the Eastern genotypes.

Supplementary Table 19. Marker-trait associations identified within genomic regions highly differentiated between Western and Eastern walnuts.

**Additional File 3**. Mass-spectrometry proteome data of catkins and pollen tissues. Three samples of each tissue type (immature catkin, mature catkin, senescent catkin, and pure pollen) were analyzed using v1.0 and v2.0 reference walnut genome assemblies. Total intensity of matching peptides, number of spectra, and percentage of protein covered by the identified peptides are reported.

giaa050_GIGA-D-19-00363_Original_Submission

giaa050_GIGA-D-19-00363_Revision_1

giaa050_GIGA-D-19-00363_Revision_2

giaa050_Response_to_Reviewer_Comments_Original_Submission

giaa050_Response_to_Reviewer_Comments_Revision_1

giaa050_Reviewer_1_Report_Original_Submission Jean-Marc Aury -- 11/27/2019 Reviewed

giaa050_Reviewer_1_Report_Revision_1 Jean-Marc Aury -- 3/24/2020 Reviewed

giaa050_Reviewer_2_Report_Original_Submission Marco Thines -- 12/16/2019 Reviewed

giaa050_Reviewer_2_Report_Revision_1 Marco Thines -- 3/23/2020 Reviewed

giaa050_Supplemental_Files

## Abbreviations

BLAST: Basic Local Alignment Search Tool; BLAT: BLAST-Like Alignment Tool; bp: base pairs; BUSCO: Benchmarking Universal Single-Copy Orthologs; BWA: Burrows-Wheeler Aligner; CABOG: Celera Assembler with the Best Overlap Graph; CCS: circular consensus sequence; cDNA: complementary DNA; CDS: protein-coding sequence; coreGF: core gene family; DTT: dithiothreitol; FDR: false-discovery rate; FLnc: full-length non-chimeric; Gb: gigabase pairs; gDNA: genomic DNA; GO: Gene Ontology; HB: haplotype block; Hi-C: chromosome conformation capture; HQ: high quality; HMW: high molecular weight; IAA: iodoacetamide; kb: kilobase pairs; LG: linkage group; LTR: long-terminal retrotransposon; MaSuRCA: Maryland Super-Read Celera Assembler; Mb: megabase pairs; MS/MS: tandem mass spectroscopy; NCBI: National Center for Biotechnology Information; ONT: Oxford Nanopore Technology; PacBio: Pacific Biosciences; PASA: Program to Assemble Spliced Alignments; RNA-Seq: RNA-sequencing; ROD: reduction of diversity coefficient; SNP: single-nucleotide polymorphism; UCD: University of California Davis; TEAB: triethylammonium bicarbonate; WIP: Walnut Immprovement Program.

## Competing Interests

The authors declare that they have no competing interests.

## Funding

This project has been funded by the California Walnut Board.

## Authors' Contributions

D.B.N. and A.M. conceived and coordinated the research. R.E.W. and W.T. performed the HMW DNA extraction and Nanopore sequencing. A.V.Z., D.P., and S.L.S. assembled the hybrid Illumina-ONT assembly. L.B., M.T., D.P.., and S.L.S. validated and anchored the HiRise assembly to the genetic maps. A.M. and B.J.A. collected and extracted all RNA samples. M.B. analyzed the PacBio Iso-Seq results and performed the repeat and gene annotation. A.D. conceived the design of the proteomic analyses; P.A.Z. and S.C. generated and analyzed the proteomic data. L.B. called the SNPs in Chandler and the 23 UCD-WIP founders, while A.M. carried out the analyses on walnut genomic diversity. E.A.D., L.B., and M.T. built and analyzed the SNP haplotypes. C.A.L. provided all the plant material. A.M. wrote the manuscript, which has been revised by all coauthors.
