# Supplementary material for: High-quality chromosome-scale assembly of the walnut (Juglans regia L.) reference genome
Source: Gigascience. 2020 May 20;9(5):giaa050. doi: 10.1093/gigascience/giaa050 (PMC7238675; doi:10.1093/gigascience/giaa050)
Supplement: giaa050_Response_to_Reviewer_Comments_Original_Submission [file giaa050_response_to_reviewer_comments_original_submission.pdf]

Author's Response To Reviewer Comments

Close

Reviewer #1: I read the manuscript by Marrano et al. entitled "High-quality chromosome-scale assembly of the walnut (Juglans regia L) reference genome" with interest. The authors describe how they generated a chromosome-scale assembly of J. regia based on ONT, Illumina and Hi-C data. In addition, genetic maps were used to validate and anchor the scaffolds to J. regia linkage groups. The authors performed a wide range of analysis, including gene families of interest and genomic diversity. The article is well written and the methods are sufficiently described.

My main concern is the quality of the assembly, although at the chromosome level, the contiguity at the contig level is ten times lower (nearly 1.1Mb) compared to the previously published Juglans genome assemblies (including J. regia).
The assembly presented in our manuscript is very high quality, with chromosome-sized scaffolds validated by genetic maps and an N50 contig size of over 1Mb.

The assembly was produced on a fairly low budget with older generation Oxford Nanopore reads from the MinION device, which yielded 21.9 Gbp with an average read size of 3.1 kb. (Today we would get reads of 10-20 Kb.) This sequencing data is older and much lower cost than those used by Zhu et al. (2019), who had 57.2 Gbp of PacBio data with an average read size of over 12kb. (see lines 136-140). Even so, a contig N50 size of 1.1 Mb is dramatically larger than our own originally published assembly contigs.

I understand that authors choose to use the methods they have developed, however, long-reads assemblies are usually made with dedicated assemblers, which can result in higher assembly quality.

Long-read-only data sets are indeed assembled with dedicated methods such as Canu, but when we have both long and short-read data sets, MaSurCA performs better. We did try using the Flye assembler, which is currently one of the best, fastest, and most accurate assemblers for long-read data, and it yielded an assembly with only 530kb N50 contig size, about half of our result.

In particular, I was a little surprised by the fact that the v2 assembly contains fewer repetitive elements than the first version of the assembly (L175-176). Generally long-reads assemblies improved the repetitive content of genome assemblies.

We re-estimated the repeat content of Chandler v2.0 by running RepeatMasker with the repeat library generated for v1. We found that 58.4% of Chandler v2 is repetitive (we have corrected the main text accordingly), which is higher than what found with the first assembly of the reference genome. Also, Chandler v2.0 is de-duplicated, meaning that almost all of the duplicated regions due to heterozygosity have been removed. These duplicates, which are variants of the same scaffold, were likely mistaken for independent scaffolds in v1.0, overestimating the genome size and, therefore, gene and repetitive contents. The de-duplication caused a reduction of the genome size from 641 Mb (v1.0) to 573.9 Mb (v2.0), which is now closer to the genome size estimate obtained with Genomescope (488.2 Mb).

The comparison of the Chandler v2 assembly with that provided by Zhu et al. is an important point for the reader, as it will determine which genome will be used for further analysis. As an example, the long-range input data are different (Hi-C vs Optical maps) and maybe specific regions are not of the same quality in both assemblies.

We compared Chandler v2.0 with JSerr_1.0 from Zhu et al (2019). We observed that more than 95% of the two assemblies aligned with sequence identity higher than 98% and exhibited high collinearity, as shown in Figure S4 (see also lines 174-179). This confirms the high quality of both assemblies, even if obtained with different technologies. The availability of both assemblies will facilitate new genomic studies (e.g., pangenome) to understand the morphological and physiological differences among these two walnut varieties better.
Chandler is the most popular cultivar of walnut worldwide. For this reason, it was chosen for sequencing

and assembling the first walnut reference genome (Martinez-Garcia et al. 2016), and is used as the standard in many genetic and biological studies (e.g., development of genotyping tools). After four years, we are here proposing a significant improvement of the reference genome. However, a detailed study of the differences between the two assemblies (e.g., structural rearrangements) goes beyond the scope of the present manuscript.

Minor Points:
* assembly and gene prediction metrics are scattered throughout the manuscript and give a descriptive tone. I think the authors can move these metrics in tables 1 and 2. In addition, contig metrics are not provided in Table 1.

We modified the gene prediction metrics, which changed due to revisions made to conform with NCBI submission requirements. We moved some of the statistics of the gene annotation in Table 2. We also added contig metrics in Table 1.

* L38: "the full sequence of all 16 chromosomes" : how is this statement validated ?

We changed the sentence to "with the 16 chromosomal pseudomolecules assembled and representing 95% of its total length" (see line 38).

* L41 and L235: Asserting that the genes are complete based solely on the presence of a start and stop codon is not enough. Please delete the term "full-length". The number of complete BUSCO genes could perhaps be a way to evaluate the proportion of full-length genes.

We removed 'full-length' and added the proportion of BUSCO genes assembled completely (see lines 269-270)

* L87 and L90: problem with the closing parenthesis.

Done.

* L97: "...walnut reference genome with unprecedented contiguity…." Please delete this sentence.

Done.

* L117: a longest read of 992.2Kb is not informative if it does not align.

We are reporting general statistics on the Nanopore sequencing. Such long reads have then been used for scaffolding Illumina reads with MaSurCa.

* L156-158: The authors should used a kmer approach (Genomescope) to estimate the genome size of both genotypes.

We ran Genomescope using Chandler Illumina pair-end reads generated by Martinez-Garcia et al., (2016). Results indicate a genome size of 448 Mb, and a level of heterozygosity equal to 0.634%. Our de-duplicated assembly has a total length of 567.2 Mb (> 1 kb), which is a great improvement compared to Chandler v1.0. However, we were unable to run Genomescope for the Serr genome since we could not find any Illumina data on NCBI. We only found 58 PacBio SRA Experiments under the BioProject PRJNA413991 indicated by Zhu et al. (2019). Also, the Illumina reads of Zhu et al. (2019) are of the hybrid 'J.microcarpa x J.regia cv Serr'.

* L239: The proportion of gene models with multiple transcript isoforms is small relative to other plants which may not represent the proportion of genes with alternative splicing. I think the low depth of PACBIO sequencing is the main reason. Please rephrase the sentence to make it clearer.

With the newly revised gene annotation, we observed more gene models with multiple transcript isoforms. We changed the text accordingly, as suggested by RW#1 (see lines 246-248).

* L269-373 : This section is not clear for non-specialist readers.

We modified the paragraph to make it clearer for non-specialist readers (see lines 275-328). We also added Figure S9 showing the different stages of catkin development considered in our analysis.

* L283 : "four developed" ?

We generated proteomes from four tissues (immature catkin, intermediate catkin, mature catkin, and pure pollen). We changed ‘developed’ to ‘analyzed’ for clarity (see line 300).

* L343 : Please describe syntelogs.

Done (line 363).

* Figure5A: There may be a problem of alignment between the inner circle and the middle circle (blue region).

We thank the reviewer for highlighting this inconsistency in the figure. We checked our data and modified the image accordingly. The apparent misalignment between the inner circle and the middle circle is actually due to a lack of information – a gap of 4.07 cM – between two haploblocks which follow one another in that region. The inaccuracy was generated by the program used to draw the image, which automatically assigned the inherited segment to the most likely ancestor based on the maximum parsimony algorithm. However, we agree that on some occasions, this can lead to inaccurate results. Therefore, we decided to explicitly represent these regions of missing information using a white color. We also amended the legend of Figure5 to explain what the white sections describe.

* Too many paragraphs end with a sentence such as "support the crucial role of Chandler v2 chromosome-scale assembly".

We removed the sentence and ended the paragraphs differently.

* L463: Please describe how the gaps have been filled.

The assembler MaSurCa used to obtain Chandler hybrid has an internal gap-filling procedure, as described in Zimin et al., 2013 (see line 476-477). In addition, Dovetail uses Illumina reads to close the gaps in the HiRise assembly, as described in lines 500-502.

Reviewer #2: Overall, this genome is a significnt advance over the previous one, but there are some points that are discussed in too much breadth, while others are too short for a detailed evaluation. Some of the claims regarding why the new genome assembly is superior over the older one(s) seem rather constructed. The parts that really profit from ONT sequencing - the near-repetitive gene families and the repeat content have not been expored in detail.

We performed a gene family analysis on Chandler’s gene predictions (v1.0, v2.0, and RefSeq). Consistently with the gene prediction results, we observed that v2.0 has, in general, more members per gene family than v1.0, and fewer members than NCBI RefSeq. These results can be due to both the increment of contiguity and the lower gene redundancy of Chandler v2.0 (see lines 254-265).

I realise that this is just a data note, but some clues could help the reader appreciate the current manuscript. What is also missing is a comparison to other published reference genomes in the Fagales s.l..

We compared assembly metrics and BUSCO percentages to other Fagales genomes (see lines 141-142; 192-193; Table S9). However, if the reviewer was referring to comparative genomics studies, then it goes beyond the scope of the present manuscript, which is a Data Note on the improvement of the walnut reference genome. Also, we already provided applications of the new Chandler assembly for proteomic and population genetics studies.

L65. How is this hybridisation possible, given the current disjuction of the populations of the species? Please give a sentence or two as explanation.

Zhang et al. (2019), cited in the text, explained clearly how the contact between American black walnuts and Asian butternuts occurred during the Pliocene. According to their reconstruction, supported by fossil evidence, butternut and black walnuts spread into Eurasia from the late Oligocene to the Miocene-Pliocene. Then, the cooling climate of the Upper Pliocene may have led to range shifts of the butternut and black walnut lineages in Eurasia, permitting the contact required for the hybridization that gave rise

to Persian walnut. We add a short sentence describing Zhang et al (2019) results in the text (see line 66).

L87. Actinida is not a tree.

We changed trees to crops (see line 89).

Ll122-125. The process of obtaining the megareads is insufficiently described. Please exapand the text and mention also the paramters used.

We added a more detailed description of how MaSurCa builds the mega-reads in the text (lines 466-478).

In addition, please provide statistics for the ONT reads and the illumina reads.

We added the statistics on the ONT reads in Table S1. The Illumina data were generated by Martinez-Garcia et al. (2016), where Illumina sequencing details and statistics are reported.

PLease also mention the library preparation technique used in both cases.

We added details on the ONT library preparation in the method section (Lines 460-464). Regarding the Illumina libraries, their descriptions are reported in Martinez-Garcia et al. (2016).

Please also metnion the known biases associated with ONT sequencing and how strong these were in your raw data.

ONT sequencing tends to have more errors in long homopolymer regions (e.g., runs of all A's). We saw no evidence that these were a particular problem in this genome, although all genomes have such regions. Our use of Illumina reads to compute the final consensus sequence for virtually all positions in the assembly should minimize this problem.

Ll126-127. How has the ols assemblly (v.1.0) integrated with the new one?

After running the de-duplication module implemented in MaSuRCA, we aligned the v1 scaffolds to v2, identified unaligned regions, and added them to v2. We added this sentence to the text (line 481-483).

L155. Has it been checked, if the unanchored small scaffold are derived from contamination with bacteria/fungi?

All assemblies have contamination, not only from the original samples but from the sequencing lab itself. We ran a thorough contamination screen, aligning every contig and scaffold against an exhaustive set of bacteria, artificial vectors, and other plants and animals, and we removed all contaminants found in this manner. In addition, NCBI runs its own contaminant screen on every submission to GenBank, and that was run on our submission as well.

L170. The identity seems rather low. The possible reasons for this sholuld be given.

We thank the reviewer for noticing this error. We estimated the average sequence identity from the nucmer coord file without filtering. In this way, we considered all alignments, including those between similar but not syntenic regions of the two assemblies. We re-estimated the percentage of sequence identity using only the 1-to-1 best alignments (command dnadiff implemented in MUMmer), and we obtain a value of 99.60. We changed the text accordingly (see lines 181-186).

L172. What was the proportion of unaligned reads? How many reads mapped discordantly?

Over a total of 432,183,992, 2,046,961 reads (0.5%) did not align and 31,169,557 did not pair properly.

L188. This statement cannot be upheld the way it is. Usually the gene space is already well-assmbled using only illumina reads (apart from the repetitive genes). The authors should compare the BUSCO scores of several Chandler assembly versions with that of other Fagales genomes, such as oak, beech, and chestnut.

We added Table S9 with BUSCO statistics for the Chandler genome assemblies, J. regia cv Serr, and other Fagales genomes.

L190. There are mapping-based ways to address this. These should be mentioned / applied.

We preferred to remove the sentence instead of following the reviewer's suggestion since revising/applying available methods for improving transcriptome assemblies using short reads is not among the scopes of our work. We aimed to prove how a much contiguous reference genome can improve transcriptome assemblies and gene predictions in walnut.

Ll217-247. This seems overly discussed, considering the rather minor differences observed.

We moved some of the statistics in Table 2 and made this paragraph less redundant and descriptive.

L363. This is not necessarily evidence of imbreeding, but could also reflect selective sweeps. Imbreeding does not happpen on the sub-genomic level but only on the genomic level.

Sub-genomic inbreeding occurs when an individual inherits the same copy of an allele at one locus from a common ancestor (identical-by-descent; IBD). Chandler parents share Payne as a common ancestor; therefore, there are high probabilities of IBD alleles at a sub-genomic level in Chandler. However, although we found no evidence of a strong selective sweep, it is also possible this pattern was due to direct or indirect selection (see lines 381-384). Future selective sweep studies in larger and more diverse walnut collections could provide more evidence on the high-level of homozygosity in some regions of the Chandler genome.

L426. Was any surface sterilisation done? Otherwise a lot of contaminant sequences would be expected.In any case, a contamination check should be reported.

We could not do surface sterilization since the Nanopore library was built starting from frozen tissue collected at UC Davis and sent to the John Hopkins University. Surface sterilization would have led to tissue degradation and plant production of stress compounds that further impede DNA extraction. Also, we ran a thorough contamination screen of our assembly, to remove contaminations associated with microbes present on the leaf surface and within the tissue (i.e., endophytes).

L428. 'g' should be in italics.

Done

L431. Concentrations/amounts missing.

Added.

L440. 'was' -> 'were'.

Done

L456. The assembly straregy, programs and parameters use are not mentioned in sufficient detail (actually hardly any of this is mentioned in the manuscript).

We added more details on the assembly strategy in the text (lines 466-478).

L531. Do not abbreviate at the beginning of the line.

Edit.

Close
