# Supplementary material for: High-quality chromosome-scale assembly of the walnut (Juglans regia L.) reference genome
Source: Gigascience. 2020 May 20;9(5):giaa050. doi: 10.1093/gigascience/giaa050 (PMC7238675; doi:10.1093/gigascience/giaa050)
Supplement: giaa050_Response_to_Reviewer_Comments_Revision_1 [file giaa050_response_to_reviewer_comments_revision_1.pdf]

Author's Response To Reviewer Comments

Close

Reviewer #1: I read the revised manuscript by Marrano et al. entitled "High-quality chromosome-scale assembly of the walnut (Juglans regia L) reference genome", my comments have been addressed and the authors modified the manuscript accordingly. I still have a few minor points that need to be checked before publication.

* I maintain that the comparison of the Chandler v2 assembly with that provided by Zhu et al. is an important point for the reader and I would expect to have a comparison of the two assemblies in Table 1.

Done

* Please check how MaSurCA is written (MaSuRCA or MaSurCa)

We named MaSuRCA consistently across the manuscript.

* Table S3 : ON instead of ONT

Changed.

Reviewer #2: I found the manuscript to be a pleasant read, with the major methodological unclarities resolved. There are two minor suggestions from my side.

1. To promte Table S9 to a full table (Table 1 and 2 could probably be condensed into one table) with the inclusion of the Quercus lobata and Quercus robur genomes, as I feel that for readers it is always good to have some upfront benchmarking so they can put the assembly into the context of other genome assemblies.

We moved Table S9 as full table in the main text (now Table 3), and we also included the BUSCO metrics for Quercus lobate and Q. rebur. We moved Figure 4 (now Figure S10) to the supplementary in place of Table S9.

2. To promote Figure S12 to a full figure (if possible).

We moved Figure S12 as a full figure (now Figure 5) in place of Figure 6 (now Figure S13).

Close
