# Supplementary material for: High-quality chromosome-scale assembly of the walnut (Juglans regia L.) reference genome
Source: Gigascience. 2020 May 20;9(5):giaa050. doi: 10.1093/gigascience/giaa050 (PMC7238675; doi:10.1093/gigascience/giaa050)
Supplement: giaa050_Reviewer_1_Report_Original_Submission — Jean-Marc Aury -- 11/27/2019 Reviewed [file giaa050_reviewer_1_report_original_submission.pdf]

Reviewer Report

Title: High-quality chromosome-scale assembly of the walnut (Juglans regia L) reference genome

Version: Original Submission **Date:** 11/27/2019

Reviewer name: Jean-Marc Aury

Reviewer Comments to Author:

I read the manuscript by Marrano et al. entitled "High-quality chromosome-scale assembly of the walnut (Juglans regia L) reference genome" with interest. The authors describe how they generated a chromosome-scale assembly of J. regia based on ONT, Illumina and Hi-C data. In addition, genetic maps were used to validate and anchor the scaffolds to J. regia linkage groups. The authors performed a wide range of analysis, including gene families of interest and genomic diversity. The article is well written and the methods are sufficiently described.

My main concern is the quality of the assembly, although at the chromosome level, the contiguity at the contig level is ten times lower (nearly 1.1Mb) compared to the previously published Juglans genome assemblies (including J. regia). I understand that authors choose to use the methods they have developed, however, long-reads assemblies are usually made with dedicated assemblers, which can result in higher assembly quality. In particular, I was a little surprised by the fact that the v2 assembly contains fewer repetitive elements than the first version of the assembly (L175-176). Generally long-reads assemblies improved the repetitive content of genome assemblies.

The comparison of the Chandler v2 assembly with that provided by Zhu et al. is an important point for the reader, as it will determine which genome will be used for further analysis. As an example, the long-range input data are different (Hi-C vs Optical maps) and maybe specific regions are not of the same quality in both assemblies.

Minor Points:

* assembly and gene prediction metrics are scattered throughout the manuscript and give a descriptive tone. I think the authors can move these metrics in tables 1 and 2. In addition, contig metrics are not provided in Table 1.
* L38: "the full sequence of all 16 chromosomes" : how is this statement validated ?
* L41 and L235: Asserting that the genes are complete based solely on the presence of a start and stop codon is not enough. Please delete the term "full-length". The number of complete BUSCO genes could perhaps be a way to evaluate the proportion of full-length genes.
* L87 and L90: problem with the closing parenthesis.
* L97: "...walnut reference genome with unprecedented contiguity…." Please delete this sentence.
* L117: a longest read of 992.2Kb is not informative if it does not align.
* L156-158: The authors should used a kmer approach (Genomescope) to estimate the genome size of both genotypes.
* L239: The proportion of gene models with multiple transcript isoforms is small relative to other plants which may not represent the proportion of genes with alternative splicing. I think the low depth of PACBIO sequencing is the main reason. Please rephrase the sentence to make it clearer.

* L269-373 : This section is not clear for non-specialist readers.
* L283 : "four developed" ?
* L343 : Please describe syntelogs.
* Figure5A: There may be a problem of alignment between the inner circle and the middle circle (blue region).
* Too many paragraphs end with a sentence such as "support the crucial role of Chandler v2 chromosome-scale assembly".
* L463: Please describe how the gaps have been filled.

Level of Interest

Please indicate how interesting you found the manuscript: Choose an item.

Quality of Written English

Please indicate the quality of language in the manuscript: Choose an item.

Declaration of Competing Interests

Please complete a declaration of competing interests, considering the following questions:

- Have you in the past five years received reimbursements, fees, funding, or salary from an organisation that may in any way gain or lose financially from the publication of this manuscript, either now or in the future?
- Do you hold any stocks or shares in an organisation that may in any way gain or lose financially from the publication of this manuscript, either now or in the future?
- Do you hold or are you currently applying for any patents relating to the content of the manuscript?
- Have you received reimbursements, fees, funding, or salary from an organization that holds or has applied for patents relating to the content of the manuscript?
- Do you have any other financial competing interests?
- Do you have any non-financial competing interests in relation to this paper?

If you can answer no to all of the above, write 'I declare that I have no competing interests' below. If your reply is yes to any, please give details below.

I declare no competing interests, however I received travel and accommodation expenses to speak at ONT conferences.

I agree to the open peer review policy of the journal. I understand that my name will be included on my report to the authors and, if the manuscript is accepted for publication, my named report including any

attachments I upload will be posted on the website along with the authors' responses. I agree for my report to be made available under an Open Access Creative Commons CC-BY license (http://creativecommons.org/licenses/by/4.0/). I understand that any comments which I do not wish to be included in my named report can be included as confidential comments to the editors, which will not be published.

Choose an item.

To further support our reviewers, we have joined with Publons, where you can gain additional credit to further highlight your hard work (see: https://publons.com/journal/530/gigascience). On publication of this paper, your review will be automatically added to Publons, you can then choose whether or not to claim your Publons credit. I understand this statement.

Yes Choose an item.
