# Supplementary material for: High-quality chromosome-scale assembly of the walnut (Juglans regia L.) reference genome
Source: Gigascience. 2020 May 20;9(5):giaa050. doi: 10.1093/gigascience/giaa050 (PMC7238675; doi:10.1093/gigascience/giaa050)
Supplement: giaa050_Reviewer_2_Report_Original_Submission — Marco Thines -- 12/16/2019 Reviewed [file giaa050_reviewer_2_report_original_submission.pdf]

Reviewer Report

Title: High-quality chromosome-scale assembly of the walnut (Juglans regia L) reference genome

Version: Original Submission **Date:** 12/16/2019

Reviewer name: Marco Thines

Reviewer Comments to Author:

Overall, this genome is a significnt advance over the previous one, but there are some points that are discussed in too much breadth, while others are too short for a detailed evaluation. Some of the claims regarding why the new genome assembly is superior over the older one(s) seem rather constructed. The parts that really profit from ONT sequencing - the near-repetitive gene families and the repeat content have not been expored in detail. I realise that this is just a data note, but some clues could help the reader appreciate the current manuscript. What is also missing is a comparison to other published reference genomes in the Fagales s.l..
L65. How is this hybridisation possible, given the current disjuction of the populations of the species? Please give a sentence or two as explanation.
L87. Actinida is not a tree.
Ll122-125. The process of obtaining the megareads is insufficiently described. Please exapand the text and mention also the paramters used. In addition, please provide statistics for the ONT reads and the illumina reads. PLease also mention the library preparation technique used in both cases. Please also metnion the known biases associated with ONT sequencing and how strong these were in your raw data.
Ll126-127. How has the ols assemblly (v.1.0) integrated with the new one?
L155. Has it been checked, if the unanchored small scaffold are derived from contamination with bacteria/fungi?
L170. The identity seems rather low. The possible reasons for this sholuld be given.
L172. What was the proportion of unaligned reads? How many reads mapped discordantly?
L188. This statement cannot be upheld the way it is. Usually the gene space is already well-assmbled using only illumina reads (apart from the repetitive genes). The authors should compare the BUSCO scores of several Chandler assembly versions with that of other Fagales genomes, such as oak, beech, and chestnut.
L190. There are mapping-based ways to address this. These should be mentioned / applied.
Ll217-247. This seems overly discussed, considering the rather minor differences observed.
L363. This is not necessarily evidence of imbreeding, but could also reflect selective sweeps. Imbreeding does not happpen on the sub-genomic level but only on the genomic level.
L426. Was any surface sterilisation done? Otherwise a lot of contaminant sequences would be expected.In any case, a contamination check should be reported.
L428. 'g' should be in italics.
L431. Concentrations/amounts missing.
L440. 'was' -> 'were'.

L456. The assembly strāregy, programs and parameters use are not mentioned in sufficient detail (actually hardly any of this is mentioned in the manuscript).
L531. Do not abbreviate at the beginning of the line.

Level of Interest

Please indicate how interesting you found the manuscript: Choose an item.

Quality of Written English

Please indicate the quality of language in the manuscript: Choose an item.

Declaration of Competing Interests

Please complete a declaration of competing interests, considering the following questions:

- Have you in the past five years received reimbursements, fees, funding, or salary from an organisation that may in any way gain or lose financially from the publication of this manuscript, either now or in the future?
- Do you hold any stocks or shares in an organisation that may in any way gain or lose financially from the publication of this manuscript, either now or in the future?
- Do you hold or are you currently applying for any patents relating to the content of the manuscript?
- Have you received reimbursements, fees, funding, or salary from an organization that holds or has applied for patents relating to the content of the manuscript?
- Do you have any other financial competing interests?
- Do you have any non-financial competing interests in relation to this paper?

If you can answer no to all of the above, write 'I declare that I have no competing interests' below. If your reply is yes to any, please give details below.

I declare that I have no competing interests.

I agree to the open peer review policy of the journal. I understand that my name will be included on my report to the authors and, if the manuscript is accepted for publication, my named report including any attachments I upload will be posted on the website along with the authors' responses. I agree for my report to be made available under an Open Access Creative Commons CC-BY license (http://creativecommons.org/licenses/by/4.0/). I understand that any comments which I do not wish to be included in my named report can be included as confidential comments to the editors, which will not be published.

Choose an item.

To further support our reviewers, we have joined with Publons, where you can gain additional credit to further highlight your hard work (see: https://publons.com/journal/530/gigascience). On publication of this paper, your review will be automatically added to Publons, you can then choose whether or not to claim your Publons credit. I understand this statement.

Yes Choose an item.
